# Organic Edible Insects—What Would It Take?

**DOI:** 10.3390/ani15162393

**Published:** 2025-08-14

**Authors:** Asia Zanzot, Emma Copelotti, Erminia Sezzi, Simone Mancini

**Affiliations:** 1 Department of Veterinary Sciences, University of Pisa, Viale delle Piagge 2, 56124 Pisa, Italy; emma.copelotti@phd.unipi.it (E.C.); simone.mancini@unipi.it (S.M.); 2 Istituto Zooprofilattico Sperimentale del Lazio e della Toscana M. Aleandri, Via Appia Nuova, 1411, 00178 Roma, Italy; erminia.sezzi@izslt.it; 3 Interdepartmental Research Center Nutrafood “Nutraceuticals and Food for Health”, University of Pisa, Via del Borghetto 80, 56124 Pisa, Italy

**Keywords:** edible insects, organic farming, animal welfare, veterinary drugs, pesticide

## Abstract

Edible insect farming represents a promising sector focused on producing sustainable and nutritional food and feed. Compared to conventionally farmed animals, insects are more sustainable, thanks to lower greenhouse gas emissions, efficient bioconversion, and minimal space requirements. These characteristics make edible insect farming remarkably eco-friendly: this concept aligns with the core principles of the organic supply chain. However, current organic regulations do not yet include insects. This review investigates various aspects of edible insect rearing to better understand how an “organic edible insect farm” could be established. Nine insect species that have been authorized as ingredients for the production of either food or feed were included, such as *Hermetia illucens*, *Musca domestica*, *Tenebrio molitor*, *Alphitobius diaperinus*, *Locusta migratoria*, *Gryllodes sigillatus*, *Gryllus assimilis*, *Acheta domesticus*, and *Bombyx mori*. Among the evaluated features of insect farming, insect welfare and the use of chemical substances (such as veterinary drugs and pesticides) are thoroughly examined in the literature review. These represent the most significant challenges given the scarce knowledge both on the well-being of insects, currently identified as non-sentient beings, and on the degradation and metabolism of drugs or pesticides that could harm the animals but also undermine consumer safety.

## Simple Summary

Sustainability is increasingly recognized as a critical global concern, given the ongoing environmental crisis. Climate change, food insecurity, and population growth are among the key factors pushing for an immediate transition towards more sustainable production systems. Organic farming is an agricultural management system that aims to maximize sustainability comprehensively, from production to distribution. Given the strong synergies between edible insects and organic farming, the integration of the former into the latter appears both logical and potentially beneficial. Edible insect farming could play an important role in the organic farming supply chain, but in order to include them in this sector, it is necessary to create regulations and guidelines suitable for this innovative production.

## 1. Introduction

The current climate situation happening worldwide is in desperate need of a change. The rapid growth of human activities is causing elevated temperature shifts and extreme weather events, which are sadly increasingly common [1]. Organic farming is defined as an agricultural method that aims to reduce environmental impact by respecting natural ecological balance [2]. Organic farming represents a way of overcoming climate change, change threats. According to the European Green Deal [3] and the Organic Action Plan [4] set up by the European Commission, the aim is to convert at least 25% of agricultural land to organic farming and increase organic aquaculture to 30% by 2030. Currently, more than 96 million hectares are managed organically by around 4.5 million farmers worldwide [5,6], while in Europe, the number of hectares grew to 17.7 million in 2023 [6]. The growth of the organic sector in Europe and worldwide is promising but may not be sufficient to meet the growing consumer demand. In fact, agricultural yields are growing slowly at 1% per year, which is considerably slower than the rapid pace at which the global population is increasing [7]. Also, organic products are growing [8] thanks to the awareness spread worldwide about the multiple advantages that organic products carry, but it seems like the production is not keeping up with the demand. A major issue regarding organic production is that organic crop yield is lower compared to conventional crops [9]. This issue has already been addressed, and it is included in the Organic 3.0 initiative [10]; however, this problem is yet to be solved. This issue leads to another critical challenge that the organic sector is struggling to overcome, which is the lack of protein meals [11]. Coherent with Regulation (EU) 848/2018 [12] on organic production and labelling of organic products, farmed animals must be fed with organic raw materials in order to be labelled as organic. The shortage of organic protein meals, such as soybean meal, represents a significant issue in the production of farm animals because it causes an increase in the prices of organic feed [11]. Also, farm animals have specific amino acid requirements that need to be fulfilled in order to obtain a qualitative product [13]; therefore, a solution to the lack of organic feeds needs to be found. In the latest years, a variety of alternative protein meals have been proposed by researchers, such as micro-algae [14], peas (*Pisum sativum* L.) [15], de-hulled expelled sunflower seeds and rapeseed [13], mussel meals [16], or edible insects [17]. The latter are a newly discovered resource that is increasingly common in the production of food and feed in Europe [18]. Despite the cultural barriers that are currently blocking the spread of this new sector worldwide [19], it is hoped that edible insects will become a useful resource both in conventional and organic production for food and feed purposes [18,20]. Indeed, Regulation (EU) 2017/893 [21] marked the first authorization of the use of insects as feed in the European Union, specifically allowing processed animal proteins (PAPs) derived from seven insect species, namely *Hermetia illucens* (black soldier fly), *Musca domestica* (common housefly), *Tenebrio molitor* (yellow mealworm), *Alphitobius diaperinus* (lesser mealworm), *Acheta domesticus* (house cricket), *Gryllodes sigillatus* (banded cricket), and *Gryllus assimilis* (field cricket) for the formulation of aquafeed. This regulatory advance was based on the positive scientific opinion issued by the European Food Safety Authority (EFSA) in 2015 [22] about the risk assessment on the use of insects as food and feed. Subsequently, insects are categorized as farmed animals (Reg. (EC) 1069/2009) [23]; therefore, they need to be fed with authorized feed ingredients listed in the Feed Catalogue of Reg. (EU) 68/2013 [24]. In 2021, the use of PAPs from the same seven insect species was extended to poultry and swine feed through Regulation (EU) 2021/1372 [25]. In the same year, the list of approved species was expanded with the inclusion of *Bombyx mori* (silkworm) as established by Regulation (EU) 2021/1925 [26]. Regarding the use of live insects as feed, this remains under the jurisdiction of individual Member States. With regard to insects intended for human consumption, in the EU, the key regulation is Regulation (EU) 2015/2283 on Novel Foods [27]. Prior to the commercialization of any insect-based product, producers must undergo a rigorous regulatory process that requires the submission of a detailed application for the product they intend to market. Authorization to produce and sell the product might undergo a scientific evaluation from EFSA, which guarantees the product’s safety. This is subsequently followed by the adoption of an Implementing Regulation, granting legal authorization. To date, insect-based products authorized for sale on the market in the EU, or requests that received a positive EFSA opinion (and are still waiting for the Commission Implementing Regulation), concern four insect species. As for *T. molitor* dried larvae [28], larvae in frozen, dried, and powder forms [29], and UV-treated powdered larvae [30] have been authorized on the market, while a positive EFSA opinion has been issued for the request about the use of frozen and dried yellow mealworm [31]. About *Locusta migratoria* (migratory locust), a single request for frozen, dried, and powdered forms [32] was authorized. Two requests about *A. domesticus* in frozen, dried, and powder forms [33], as well as partially defatted cricket powder [34], were authorized, while two are still pending for the implementing regulations [35,36]. Finally, one request for *A. diaperinus* larvae in frozen, paste, dried, and powder forms [37] was authorized. Moreover, there are several pending applications that are still waiting for approval, regarding various insect species, such as *H. illucens*, *Apis mellifera*, *L. migratoria*, *A. diaperinus*, *T. molitor,* and *Gryllodes sigillatus*. Legislatively, these regulations serve as the fundamental basis for the production of insects as food and feed. What remains absent to date is a regulatory framework or guidelines detailing the procedures for organic insect rearing. Sustainability is the key factor that links organic farming with edible insect production. The heart of Europe’s production policy is a strategy that aims to make food systems fair, healthy, and environmentally friendly, and this concept is fiercely pursued by organic farming. Edible insects represent a sustainable choice in the animal production sector, especially when compared to conventional farm animals. Sustainability, as intended by its definition given by the Food and Agriculture Organization of the United Nations (FAO) [38], should comprise not only the environmental aspects but also consider economic and social factors. Therefore, a sustainable production should be eco-friendly, profitable, and give benefits to society. Edible insect production aligns well with these concepts. This new “mini-livestock” sector [39], according to various studies concerning different species, has a lower environmental impact in terms of emissions of methane, nitrous oxide, and carbon dioxide [40,41], but also concerning deforestation, soil erosion, and desertification [42]. As reported by Oonincx and de Boer [41], to produce 1 kg of protein, mealworms need 90% less land when compared to beef. Also, in terms of water use, insects require a minimum quantity to be reared: they are usually given agar-agar cubes, vegetable slices as a source of water, or alternatively, the humidity rate of the substrate is sufficient [43,44]. Another substantial strength that characterizes edible insects is their ability to feed on a wide variety of organic waste, ranging from vegetable remains to animal manure. In this optic, following the concept of circular economy, all the waste coming from the organic agriculture or livestock farming could be reused to rear insects. Insects also have a potential socio-economic influence [45]. The birth of an organic edible insect sector would represent a new economy that would bring the industry new job opportunities, improving the livelihoods of many families and alleviating poverty [46]. Furthermore, insects could contribute actively to reaching Goal 2 of the Sustainable Development Goals [47], “eradicate hunger,” as they represent a good nutritional source [48] and they are easy and cheap to rear [49]. One of the key differences between conventional and organic livestock farming is the approach to animal feeding. In organic farming, in compliance with Reg. 848/2018 [12], animals must be fed with organic ingredients and raw materials in order to be included in the organic supply chain. As for edible insects, which are currently not included in the latter regulation, a major characteristic that emphasizes the sustainability of their production is their ability to grow on a wide variety of organic wastes, side streams, or byproducts [17]. The use of these materials, if properly monitored, would not only help reduce the quantity and impact of global waste but could also serve as a novel, environmentally neutral, and nutritional alternative for food and feed purposes. If insect farmers had to follow the current regulation of organic production, they would be obliged to feed their insects with organic materials. However, this could cause competition issues considering the lack of organic raw materials—especially proteins. As reported above, given the current regulation on farmed animals feeding (Reg. (EC) 1069/2009 [23] and Reg. (EU) 68/2013 [24]), the remarkable ability of insects to upcycle organic waste into valuable biomass could not be fully exploited. Nevertheless, the Final Report on Insect Production for Food and Feed emitted by the Expert Group for Technical Advice on Organic Production (EGTOP) [50] recommends that at least 95% of the nutritional input for organic insects comes from (conventional) side streams regionally sourced, of which 60% and 25% should be organic, respectively, for food and feed purposes. This approach aligns with the horizontal framework for organic production while also fully harnessing the potential of insects, thus enhancing the overall sustainability of the sector. The possibility of using conventional waste and side streams [50] could pose a risk in terms of contaminants that may be present in quantities within the Maximum Residue Limits (MRLs) posed by the EU. These could represent a threat to insects’ health; therefore, it is fundamental to assess and evaluate their effects. Another aspect currently overlooked in the edible insect farming sector is slaughtering. In Europe, Regulation (EC) No 1099/2009 [51] on the protection of animals at the time of killing aims to minimize unnecessary stress and suffering. However, this regulation does not currently apply to insects, as they are generally considered non-sentient beings—thus incapable of experiencing emotions or pain.

What can an insect feel? This is the main question that lingers around farmers and researchers. This inquiry first arose in 1984 when Eisemann et al. conducted a study on a locust that was feeding while simultaneously being eaten by a praying mantis [52]. Researchers concluded that, considering the role of pain, the neural organization of insects, and their behaviour, insects are not capable of feeling pain, at least not in the same way as vertebrates. Opinions on this topic are diverse and often contradictory, with discrepancies still to be clarified. Invertebrates are currently not considered sentient beings (with the exception of cephalopods); therefore, they are considered unable to feel pain or stress [53,54]. This concept does not raise concerns within the public opinion due to the fact that invertebrates—especially insects—are considered pests, and they generally create fear and aversion between people; therefore, their welfare is not currently considered as an issue [53]. Nevertheless, given the importance that the organic production chain places on animal welfare, this issue will certainly need to be reconsidered in the context of establishing a certified organic edible insect sector. In addition, several studies have demonstrated that the method of slaughter can affect the characteristics of the final insect-based product [55,56,57,58]. However, none have specifically focused on identifying a fast and potentially painless method to minimize stress and suffering in insects at the time of slaughter. In current farming practice, insects are mainly slaughtered through blanching, freezing, crushing, or grinding. These methods are recommended by the International Platform of Insects for Food and Feed (IPIFF) and, more recently, in the final report of the EGTOP, as they are considered faster and less brutal compared to alternative methods such as sand roasting, microwaving, freezing in air, and asphyxiation.

So, how can edible insects be included in organic production in the European Union? As of now, given the lack of EU regulations regarding an organic edible insect sector, every aspect of this agricultural system must be analyzed and insect-oriented. Considering the affinity already present between the two, it is likely that soon researchers will start focusing more and more on finding organic adaptations for this new sector.

In this review, various macro-areas will be analyzed to intersect organic farming concepts with the edible insect production, such as sustainability, feeding, animal welfare, and the use of chemical substances, to understand what it would take to create a brand-new organic edible insect sector.

## 2. Materials and Methods

The methodology adopted in this study is a narrative review of the current scientific literature. The literature search was conducted between March and May 2025 within Google Scholar and PubMed databases. All the selected studies were published in peer-reviewed journals through the Web of Science online database. We comprised nine insect species, selected based on their approval as ingredients in food (Reg. (EU) 2021/882 [28]; Reg. (EU) 2021/1975 [32]; Reg. (EU) 2022/169 [29]; Reg. (EU) 2022/188 [33]; Reg. (EU) 2023/5 [34]; Reg. (EU) 2023/58 [37]; Reg. (UE) 2025/89 [30]) or feed (Reg. (EU) 2017/893 [21]; Reg. (EU) 2021/1372 [25]; Reg. (EU) 2021/1925 [26]) in the European Union; therefore, *Hermetia illucens*, *Musca domestica*, *Tenebrio molitor*, *Alphitobius diaperinus*, *Acheta domesticus*, *Gryllus assimilis*, *Gryllodes sigillatus*, *Locusta migratoria* and *Bombyx mori* were included. As for the search strings, we used both the common name and the scientific name of the selected species together with in-quotation marks “edible” and keywords on the addressed topic. The latter were “stress” for the Insect Welfare section, “veterinary drugs” and “antibiotics” for the Veterinary Drugs section and “pesticide”, “insecticide” and “herbicide” for the Pesticide section (e.g., “Edible” Black soldier fly “stress” or “Edible” *Locusta migratoria* “insecticides”). This work focuses on insects for food and feed purposes; therefore, articles addressing insects as pests were not included.

Our research was restricted according to the following criteria: (1) short communications, theses, abstracts, reviews and books were not included; (2) only English-written articles were chosen; (3) there was no restriction on the authors’ nationality; (4) no restriction was set as for the year of publication.

## 3. Insects’ Welfare

Public concern about animal welfare has notably grown over the last century, leading most consumers to choose food products coming from ethically responsible farming [59]. Animal welfare is a key element in organic farming. According to IFOAM, farmers of the organic supply chain put the well-being of their animals first by allowing their natural behaviour, preserving their health and the balance of their environment, providing them a general good care. As stated by Reg. (EU) 848/2018 [12], farmers must preserve animal welfare throughout the entire rearing period, both in terms of physical, physiological and ethological points of view. At present, in insect rearing facilities, as well as on a laboratory scale, there is no legislation that can be applied to guarantee insect welfare. In a potential organic edible insect facility, farmers should follow the instructions given by the organic regulation to be labelled as organic. Animal welfare is not only a crucial concept per se, but it is also closely linked to various other factors that work together to create a more sustainable and well-rounded farm. A well-fed, healthy animal, free of environmental stresses or fears, is a more productive animal; therefore, it is in the interest of farmers to prioritize the well-being of their animals in order to enhance overall yield [60]. However, in the latest years, researchers—but also farmers—have started to be concerned about this topic, due to the uncertainty surrounding this issue [61]. In fact, the latest theories are emerging, stating that some insect species may be sentient [62]. Insects are confirmed to have nociceptors. However, it is crucial to make a distinction between pain and nociception: the latter is intended as the ability to detect harmful and potentially damaging stimuli [63] and is not directly related to pain. It has been demonstrated that insects exhibit “nocifensive behaviours” [64], prompting the question of whether they can experience pain. Following these concerns, many researchers suggest that the precautionary principle should be applied [54]. As the precautionary principle declares, insects should be treated as sentient beings in order to avoid them any pain or discomfort [65]. Given the limited understanding of insect welfare, the majority of farmers attempt to implement Brambell’s Five Freedoms [66] within their facilities to ensure the well-being of their animals, an application that is also promoted by IPIFF. The freedom of discomfort, for instance, requires providing an appropriate environment that meets the physiological needs of insects [67]. Given the considerable variability among insect specimens and the uncertainty surrounding the definition of an “adequate environment”, the application of this concept is not straightforward [68]. As a result, this causes the lack of a standard welfare framework that could be applied in insect farms, and farmers act in different ways according to their preferences [68]. Various researchers are exploring more in-depth insect physiology and behavioural characteristics to better understand how to apply insect-friendly practices in facilities and lab-scale systems. To be able to minimize stress during the farming of insects, major characteristics to be concerned about are practical aspects of rearing, such as temperature, rearing density, feed availability, and handling. In nature, insects may experience other types of stressful events, such as encounters with predators. Even though handlers could be perceived by insects as a kind of predator, predator-induced stress does not encompass the farm environment; further information about this kind of stressful event can be found in the review by Cinel et al. [69]. Many of the studies focused on environmental or artificial factors that could impact farmed insects are more concerned about the yield rather than the well-being of animals; therefore, further investigations are needed. As already mentioned, welfare is closely related to production yield; thus, engaging in practices that can respect insects’ well-being will result in a more profitable production. Additionally, it has been demonstrated in multiple works that stress can induce an increase in phenotypic and genetic variation in quantitative characters, especially those linked to fitness [70,71]. In Table 1, Table 2 and Table 3, scientific publications on the effects of different stresses and their detection are listed. In these research papers, scientists evaluated insects’ reactions to artificially applied stressful stimuli in order to examine the responses/effects. The stressors employed vary from temperature stress to feed deprivation and others. A cluster of different reactions has been reported, and most of them affected not only the productivity of insects but also their fitness traits, meaning that the event was considered stressful. Thermal stress—both applied with high and low temperatures—is generally the most tested (Table 1). Temperature is a key factor in insect rearing, given that most of the production species have an optimal thermal range that should be maintained in order to provide the best environment to ensure insects’ well-being, but also to optimize production. Authors who investigated the effects of high and low temperatures used different methods of evaluation, such as growth and survival rates, reproductive capacity, locomotor activity, nutritional composition, and nutrient assimilation and conversion. Insects require relatively high temperatures to be reared, but when it gets too hot (over 35 °C), it can undermine insects’ growth, survival rates and mortality rates [72,73,74,75,76,77,78,79,80,81,82], but also their nutritional composition [83,84,85] (Table 1). This aspect affects the insects differently in relation to the species and the life stage. In rare cases, exposure to higher temperatures can have positive effects on production traits, although it could imply putting animals in a stressful situation. For instance, Herren et al. [86] stated how a short heat shock in *Tenebrio molitor* larvae determined higher survival rates in individuals exposed to the fungal pathogen *Metarhizium brunneum* and increased their haemolymph antibacterial activity. Similarly, cold temperatures can cause the same detrimental effects, leading to a decrease in survival rates, delays in growth, and maturation times [73,80,84,87,88,89,90]. To understand the critical threshold for high temperature, heat knockdown time (HKDT) can be used to measure heat tolerance in insects. Heat knockdown time is defined as the period between the first exposure to elevated temperatures until the incapacity of the individual to stand upright [91]. This parameter is influenced by a variety of factors, including life stage [92], body size [93], food availability [94], and many others. Insects have a particular ability to cope with extremely hot temperatures, which is the formation of heat shock proteins (HSPs) that can prevent cell damage [95]. Heat knockdown time and HSPs synthesis are triggered by exposure to elevated temperatures, and various researchers have focused on their characterization, which, according to the literature, can be influenced by the intensity of the temperature, duration of exposure, sex, type of tissue, and acclimation [83,96,97]. Moreover, many insect species are also supplied with complex physiological and biomechanical adaptations that protect them from extreme cold temperatures [98]. Based on these mechanisms, insects can be divided into the following two categories: freezing-tolerant species and freezing-intolerant species. The former can overcome extracellular ice formation, while the latter have a breaking point under which they are not able to fight cold temperatures and succumb. This limit is called the supercooling point (SCP), and in intolerant species, it may correspond to the absolute lower lethal temperature [99]. Above the SCP, insects’ bodily fluids do not freeze; therefore, it gives them a major resistance to cold [100]. In this regard, Salin et al. [101] investigated the SCP in adults and larvae of *Alphitobius diaperinus* when subjected to starvation. Results state that the SCP of starved adults was lower than the SCP of fed adults, with a marked effect on males compared to females. Although SCP is notably affected by many other factors—e.g., polyols, water content and elimination of internal nucleation sites in the digestive tract [101]—Salin’s findings show that starvation and sex also contribute to lowering the SCP of *Alphitobius diaperinus* adults. Moreover, *Tenebrio molitor* larvae have also shown equal methods of protection from cold [102]. In the same manner as heat knockdown time, chill coma recovery time (CCRT) is also used to determine insect resistance to cold, and it is defined as a state of temporary paralysis due to low temperatures [103]. This measure is often used to determine the temperature under which an insect’s ability to survive and function is compromised. Prolonged exposure to lower temperatures not only extends the duration of chill coma but also significantly reduces the likelihood of a full recovery upon re-establishing warmer temperatures. Individuals exposed to such conditions frequently exhibit both physical and physiological impairments that compromise fitness performances and ultimately reduce survival rates [104]. Moreover, chill coma recovery time seems to be affected by other factors, such as sex, both hot and cold acclimation, feed availability, and their interaction [75,87,105,106,107]. Furthermore, together with HKDT and CCRT, two other tools used to investigate insects’ thermal tolerance and range are critical thermal maximum (CTmax) and critical thermal minimum (CTmin), defined as the upper and lower temperatures at which an organism loses the ability to perform coordinated activity [108]. When exposing an individual to thermal stress, the use of these four measures can efficiently assess the physiological limits within which the individual is able to function properly and without permanent damage. Clearly, the thermal range of insects can be influenced by a wide number of physiological factors, such as life stage, species, sex [109,110], but also environmental elements, namely temperature, acclimation, relative humidity rate, availability and quality of nutrition sources [109,111]. Another aspect that is affected by extreme temperatures is insects’ reproductive activities [76,79,112,113,114,115]. High rearing temperatures enhance the egg-laying rates of different species, but it should not get too hot. Taking *Tenebrio molitor* as an example, various studies over the years have established its optimal temperature range to be between 25 and 30 °C [116]. However, according to Morales-Ramos et al. [117], reproductive performances of this species—fecundity, egg viability, and larval development—reach their peak at 27 °C. An increase in temperature of just 4 °C—up to 31 °C—appears to be detrimental to the reproductive performance mentioned above. This means that even a slight rise can compromise the performance of this species and, consequently, cause stress and impact its well-being. In *Acheta domesticus* male crickets, cold exposure (0 °C for 6 h) caused an inability to produce a spermatophore; therefore, they were unable to mate (did not show any courtship behaviour). Nevertheless, subsequent contact (>1 h) with females could restore their reproductive capacity, thus cold stress does not affect crickets’ fecundity [114]. Thermal stress can also affect the locomotor activity of insects, and it was reported in several studies [77,105,118,119]. Schou et al. [118] describe how an increase in temperature (up to 35 °C) combined with different densities caused lower activity in *Musca domestica* flies. Likewise, Kjærsgaard et al. [77] showed how different *Musca domestica* fly populations exhibited reduced activity over time with the increase of temperature (up to 43 °C). Additionally, Robertson et al. [120] reported how a heat shock event slowed the decrease in wing beat frequency during sustained flight in *Locusta migratoria*, while in normal conditions, wing beat frequency gradually decreases in long-term flights. Extreme temperatures can affect a wide variety of aspects, not only the ones cited above. In the literature, many other aspects in various insect species have been investigated, such as morphogenetic consequences after exposure to thermal stress [121], immune responses and haemocytes count [86] and catalase activity [122]. Besides temperature, insects require appropriate humidity rates in order to grow, although humidity influences their development to a lesser extent compared to temperature [123,124,125,126,127]. Nevertheless, humidity alone plays a role in insect rearing if not associated with correct temperature rates, but adopting inadequate humidity rates can cause severe problems in insect farming. As described by Punzo and Huff [123], for *Tenebrio molitor* and Tenebrio obscurus larvae, dry conditions (12% RH) generally represent a stressful situation for larvae, pupae, and adults when compared to mesic conditions (73% RH). Dry conditions also seem to affect the embryonic development of eggs of the latter cited species, regardless of the temperature. In a work with *Hermetia illucens* larvae and adults, Holmes et al. [125] stated that low humidity rates (25% RH) caused higher mortality in eggs, larvae and pupae, but also elongated developmental times, decreased adult emergence rates and adult longevity. In terms of larval development, Johnsen et al. [127] demonstrated that relative humidity has a significant impact on the growth of *Tenebrio molitor* larvae. Notably, despite these differences in body mass and length, survival rates were not affected by the variation in humidity rates. Insects are also affected by photoperiod, and in the literature, some studies about light effects—both in terms of visible light and in intensity—can be found [128,129,130]. According to Ferenz’s [129] work, the viability of the *Acheta domesticus* egg is strongly influenced by coloured light. Talking about light, Cattaneo et al. [130] evaluated volatile compounds (VOCs) emissions by *Hermetia illucens* larvae while subjected to double stress situations, namely the presence/absence of light and purging. Although the trial did not show any statistical differences, the analysis of VOCs showed differences in compounds emitted by larvae. Some of the compounds released in the light treatment are usually identified as stress compounds (2,4-dimethyl-1-heptene, 1-cyclopentylethanone and hexadecane); therefore, purging in light can represent a stressful situation for black soldier fly larvae.

Availability of resources—both feed and water—can also cause issues in reared insects [84,89,128,131,135,136,137,138] (Table 2). Food starvation or dietary restrictions may lead to a consumption of energy reserves, and this could be worsened when combined with other types of stressors, such as extreme temperatures. In this regard, Renault et al. [131] proved how starvation (for 35 days) negatively affected the body mass of *Alphitobius diaperinus* beetles, regardless of temperature, while in fed beetles, temperature had a significant effect on their weight. In addition, after 35 days of starvation, beetles had low survival rates (13, 14 and 13%) despite the differences in rearing temperatures (12 °C, 20 °C and 24°C) (Table 1 and Table 2). Fasting can also affect shelter use and grooming activities in *Acheta domesticus*, according to Vossen et al. [138]. Vossen demonstrated how, after only 24 and 48 h of fasting, crickets spent more time inside a shelter, while the duration of time spent in an exposed area decreased significantly. During the fasting period, crickets also performed fewer grooming activities. These behaviours can be described as a strategy to preserve energy when facing a stressful situation like food deprivation. Water deprivation can also result in growth issues. Dehydration can lead to a depression in biomolecular growth, therefore reducing physiological performance. Indeed, McCluney and Date [139] report higher dry mass and body length in *Acheta domesticus* crickets with higher hydration levels compared to those with lower hydration.

**Table 2:** Effects of the availability of resources (food and water) on edible insects.

Order	Specie	Stress	Evaluation	Stage	Effects	Reference
Dip	*Hi*	F L	Volatile organic compounds (VOCs)	L	Changes in VOCs emission (no statistical differences)	[130]
Col	*Adi*	F T	Metabolic responses	A	>survival at low t° during starvation Non-F beetles: <water content at 12–16 °C 12 °C: <triglycerides and glycogen in non-F beetles <triglycerides, glucose, glycogen and protein; >glycerol in F beetles 16–20 °C: <triglycerides, glycogen, glycerol, glucose, protein in F beetles	[131]
Col	*Adi*	F T	Survival O_2_ consumption	A	Fed beetles: >O_2_ consumption >t°, >O_2_ consumption	[89]
Col	*Adi*	F T	Survival ratio Free amino acid levels	A	Longer survival for females >free amino acids with C	[84]
Ort	*Ado*	W	Hydration state Growth	A	>hydration, dry mass and length with 24 h water availability	[139]
Ort	*Ado*	F	Shelter use	A	>shelter use with longer F <grooming behaviour with longer F	[138]
Ort	*Ado*	F L	Haemocyte count Protein content Body weight	A	16 L:8 D, >haemocytes in males 12 L:12 D, >haemocytes in females <haemocytes in females with F 16 L:8 D, <protein content in haemolymph	[128]
Ort	*Gs*	F	Offspring fitness	A O	F in female crickets: >eclosion time of O <O survival to sexual maturity <body weight of O	[135]
Ort	*Gs*	F	Asymmetry (FA) Reproductive performances	A	<food quality, >FA, <reproductive performances	[136]
Lep	*Bm*	F T	Thermal tolerance Chill Coma recovery time (CCRT)	L	F, <CCRT F + Heat shock, minimum CCTR	[106]

Dip: Diptera; Col: Coleoptera; Ort: Orthoptera; Lep: Lepidoptera; *Hi*: *Hermetia illucens*; *Adi*: *Alphitobius diaperinus*; *Ado*: *Acheta domesticus*; *Gs*: *Gryllodes sigillatus*; *Bm*: *Bombyx mori*; T: thermal (stress); F: fasting; L: light (stress); L: larva; A: adults; O: offspring.

Another type of stress that edible insects may encounter in rearing conditions is mechanical stress (Table 3). Mechanical stress refers to any kind of action that determines manipulation—direct or indirect—of animals within their rearing environment. Insects in nature are not used to being manipulated by farmers; therefore, this can cause stress. Handling, tumbling, and sieving are the most frequent activities that can be included under mechanical stressors. Research on this topic is still limited, but some authors have investigated physical and physiological effects on insects [107,140,141]. Orchard et al. [140] have explored octopamine content in *Locusta migratoria* after experimental handling, and they have discovered that octopamine levels in haemolymph rise after locusts are manipulated. Octopamine is a neurohormone belonging to the group of biogenic amines widely present in insects. It has been shown that an increased level of such a molecule is determined by a wide spectrum of factors, which includes basic daily events, such as moulting or physical activities [142], but also infections and unpleasant conditions, which makes octopamine known as the “stress hormone” in invertebrates [143]. In the literature, many other stressors and physiological responses are tested in various edible insect species, such as anoxia and CO_2_ exposure [124,144,145], rearing densities [146,147,148], metal and chemical stress [132,149,150,151,152], ingestion of noxious material [153,154] and the interaction of different stressors (Table 3). Ultimately, there are multiple stressors that can negatively affect the farming and performance of edible insects. Similarly to conventional livestock, abiotic conditions, such as temperature, humidity, air quality, and photoperiod, within the rearing environment are fundamental factors to be carefully controlled in order to ensure optimal conditions for the animals. Likewise, it is essential to consistently provide necessary resources such as feed and water, which must be of appropriate quality and free from contaminants, in order to prevent unnecessary stress in the insects. Quality control of substrates intended for insect rearing is often underestimated, yet it is of considerable importance, particularly when such substrates are derived from waste or by-products, which are more likely to contain undesirable substances. Despite the lack of knowledge about insects’ welfare, many researchers have already analyzed aforementioned stressors and consequent reactions of insects, but further research is needed in order to better understand how insects’ nociception works, therefore enhancing rearing conditions for this type of production.

**Table 3:** Effects of multiple environmental stressors (mechanical stress, density, gas exchanges) on edible insects.

Order	Specie	Stress	Evaluation	Stage	Effects	Reference
***Mechanical stress***						
Col	*Tm*	Mechanical irritation Disturbance of gas exchanges	Calorimetric investigations	P	>exothermic peaks with disturbance of CO_2_ exchanges and mechanic pulsations	[141]
Ort	*Lm*	Experimental handling	Octopamine level in haemolymph and lipid content in haemolymph	A	>octopamine levels and lipids in haemolymph	[140]
Ort	*Ga*	Manipulation Implantation	Survival Oviposition rate Ovarian mass and egg number Area and length of laid eggs Hatching vigour Food consumption	A N	Implanted crickets: >mortality, <oviposition rate <E area from implanted crickets and <E length <vigour in implanted N <consumption in implanted females	[107]
***Density***						
Dip	*Md*	D H	Locomotor activity	A	>D, <activity >t°, <activity	[118]
Col	*Adi*	D	Overall larval biomass Development time Larval growth Feed consumption metrics	L	High D, >overall biomass High D, >development time, <larval growth High D, <feed consumption metrics	[148]
Ort	*Ado*	D	Growth Survival Starvation resistance	N	>D, >non matured crickets, >growth rate in females >mass in females at <densities after F >days of survival in males at <D	[147]
Lep	*Bm*	Solitary living	Body weight Ingestion	L	Solitary living: <excretion, <ingestion, <body weight, >agility	[146]
***Chemical stress***						
Col	*Adi*	Ch T	Recovery time Active Larvae	A	Recovery time based on population and duration of exposure to extreme t° <number of active L with insecticide <probability of recovery with insecticide	[132]
Col	*Tm*	Metal	Anti-Freeze Proteins (AFPs) production	A	<AFPs production	[150]
Ort	*Gs*	Microplastic ingestion	Growth rates	A	<size/weight in females with >concentration of polyethylene terephthalate microfibers	[154]
Lep	*Bm*	Ag nanoparticles ingestion (AgNPs)	Body weight Fat body protein spots	L	Ag nanoparticles ingestion, >body weight >concentration AgNPs = death Differences in fat body protein spots	[153]
Lep	*Bm*	Polystyrene nanoparticles	Locomotor activity Development	L	Erratic movements Interference with feeding initiation	[119]
Lep	*Bm*	Chromium (Cr) exposure	Gene expression Survival rates	L	>exposure, <survival Different gene expression >cellular stress and toxicity mechanisms and >defence against oxidative stress Downregulation of the folate biosynthesis pathway with high Cr exposure	[152]
***Gas stress***						
Dip	*Md*	T RH G	Calorespirometric investigations	P	>t°, <development	[124]
Col	*Tm*	Mechanical irritation Disturbance of gas exchanges	Calorimetric investigations	P	>exothermic peaks with disturbance of CO_2_ exchanges and mechanic pulsations	[141]
Col	*Tm*	G	Growth rate Larval size N° of moults Sex ratio of pupae Mortality Development abnormalities	L	<O_2_, >moults >development time at <O_2_ <O_2_, <growth rate <O_2_, >mortality <O_2_, >A abnormalities <O_2_, >surviving females	[145]
Ort	*Ado*	Anoxia CO_2_	Mortality Feeding Growth Metabolism Water balance Blood composition	N	>exposure time, >mortality, >extension of instars, >weight loss >drinking inhibition at any exposure level <food consumption, >metabolic rate reduction >weight loss, <blood volume, >lipid in blood, <blood pH	[144]

Dip: Diptera; Col: Coleoptera; Ort: Orthoptera; Lep: Lepidoptera; *Md*: *Musca domestica*; *Adi*: *Alphitobius diaperinus*; *Tm*: *Tenebrio molitor*; *Lm*: *Locusta migratoria*; *Ado*: *Acheta domesticus*; *Gs*: *Gryllodes sigillatus*; *Ga*: *Grillus assimilis*; *Bm*: *Bombyx mori*; RH: relative humidity; T: thermal (stress); H: heat (stress); G: gas (stress); D: density (stress); Ch: chemical (stress); L: larvae; N: nymphs; P: pupae; A: adults.

## 4. Veterinary Drugs

In conventional livestock farms, antimicrobial agents have been widely used since the 1950s to treat infections in sick animals, but also to promote growth and as a preventive measure [155]. This generated a worldwide concern due to the presence of drug residues in animal-derived products, but also in manure, and consequently in soil and water. As reported by Van Boeckel et al. [156], around 73% of antibiotics are used in food-producing animals, and this uncontrolled spread of antibiotic residues has been contributing to the antibiotic resistance issue. This is considered a major global health crisis, since pathogens are becoming increasingly resistant to conventional antibiotics [157]. In compliance with Regulation (EC) 1831/2003 [158] on feed additives, antibiotics have been banned from being used as growth promoters since 2006. As the regulation previously cited states, antimicrobials are now used just as treatment, but still, the antibiotic-resistance issue is yet to be solved [159]. In the organic farming regulation [12], the use of chemically synthesized allopathic medicines is strictly limited. As the regulation states, the preventive use of antibiotics in organic livestock is forbidden, even if these substances can be used to treat a sick animal. In this regard, in compliance with Reg. 2018/848 [12], the withdrawal period of such animals is doubled and should last at least 48 h. In organic farming, the preferred path to avoid health conditions and prevent diseases is to guarantee optimal hygiene standards, combined with correct feed regimes and good husbandry practices. Also, organic farmers should choose suitable breeds that align with the organic farming principles. These breeds should be able to adapt to outdoor conditions, and farmers should take into consideration their resistance to diseases, their vitality, their natural hardiness and grazing ability [160]. Edible insects, like any other farmed animals, can be affected by diseases, infections, and parasites, leading to high mortality rates and a decline in health status [161,162]. Insects can come into contact with a wide variety of pathogens, including viruses, bacteria, fungi, protozoans, nematodes, and with the rise of large-scale production for food and feed at higher stocking densities, the risk of disease outbreaks is increasing [161,163]. Indeed, in the literature, there is evidence of insect rearing systems being attacked by pathogens, in particular, beekeeping and silk farming [164,165]. Despite the natural defences that insects are equipped with, if a disease were to spread out, farmers—of both conventional and organic facilities—are authorized and obliged to treat animals by administering veterinary drugs or appropriate treatments on behalf of insect welfare. Indeed, insects can be treated with medicines or antibiotics, just like any other farmed animal. Given the lack of information about insect diseases and how to treat them, farmers often underestimate potential disease outbreaks, but with the growth of the edible insects farming sector and the potential risks of zoonosis and transmission of pathogens in the environment, things have to change. As reported by Maciel-Vergara et al. [165], symptoms can concern both production traits and physical appearance. Flaccidity, colour changes, and bad odour are usually the first signs of bacterial diseases, but some may cause characteristic symptoms, which are more difficult to recognize and detect. The current way to combat these issues is to work in prevention and apply biosecurity measures, just like any other animal farm. Notably, farmers should avoid stressful situations, such as sudden changes in temperature/humidity rates, dietary changes, nutritional deficiencies or overcrowding, to enhance insects’ immune system and prevent health problems. While these may seem like obvious measures for welfare implementation on farms, they currently represent the primary and, in many cases, the only way of safeguarding insect health. It is therefore crucial that farmers strictly adhere to these practices. Prevention is mandatory, but still, there is a need to better understand how to treat ill insects without compromising the healthy ones. Usually, when signs of a disease appear in a colony, it is difficult to stop the spread of the illness due to the high densities of insects in small enclosures, and consequences can be severe. Moreover, for the same reasons cited above, it is complicated in the first place for operators to detect visible clinical symptoms. In this regard, insect disease management could hypothetically resemble the situation happening in conventional poultry farming. The high rearing densities usually applied in poultry farming lead to higher risks of disease outbreaks, diseases that can rapidly spread throughout the entire colony, resulting in huge economic losses for the farmers and safety concerns for consumers [166]. To address this issue, a widespread approach is to treat the whole flock instead of the ill individuals [167]. Similarly, taking into account the reduced body size of insects and their high stocking densities, it is much easier to treat the whole colony as a single unit rather than treating only the sick individuals. At the same time, there is an urgent need to better understand clinical symptoms of insect diseases in order to detect their signs as early as possible, thus reducing damage to the colony. Moreover, considering the present state legislated by the organic regulation, a collective treatment would not be permitted, given the set limitations for the use of chemically synthesized allopathic drugs. A more drastic solution would be the complete eradication of the colony and subsequent sanitization of the rearing environment, but this would represent a huge economic loss for the farmer. Another important aspect of using veterinary drugs—especially antibiotics on farmed insects is the withdrawal period. As withdrawal period is intended to be the time between the administration of the pharmacological therapy and the possibility of reintroducing the animal into the production chain. Furthermore, in organic farming, animals should not exceed a certain number (species-specific) of treatments over their productive life; if the limit is exceeded, the individual may be subjected to exclusion from the organic supply chain. The number of treatments in their lifetime and the suspension period can vary based on the species and their lifespan, the drug dosage, the weight of the animal, and the type of drug. As of now, information regarding insects’ ability to react to and metabolize veterinary drugs is scarce; therefore, it is difficult to determine a specific withdrawal period, as well as the correct dosage for treatment. The maximum number of treatments an animal can undergo depends on the length of its productive lifespan. Since insects have a short life cycle, further research is required in order to determine appropriate treatment strategies. In Table 4, studies on the use of veterinary drugs on edible insect species are listed. Researchers focused on both the effects on growing and development parameters, reproductive capacity and, in some cases, residues of drugs in insects and substrates after rearing. Based on the type of administered drug, dosage, and way of inclusion—inoculated in the substrate or injected into the live animal, insects can react in various ways, both with negative and positive responses. Some literature sources reported better weight gains in insects treated with antibiotics, higher survivability and a lesser incidence of diseases [168,169,170,171]. In 2016, Lalander et al. [170] analyzed the effects of a mix of three pharmaceuticals added to *Hermetia illucens* larvae feed. After 27 days of rearing in spiked substrates, larvae reached a higher mean weight than control larvae (225 mg vs. 214 mg). Moreover, better rearing performances of silkworm were reported in other studies about the use of antibiotics [168,169,171]. On the other hand, many sources reported negative performances of insects reared on drug-contaminated substrates when compared to control diets, both in terms of growth and development, but also of survival rates and overall production yields [172,173,174,175,176,177,178,179,180,181,182,183,184,185,186,187,188,189,190,191]. Different outcomes can be related to the employed dosage of veterinary drugs and antibiotics on animals. This was demonstrated by Gao et al. [181], where they administered three different dosages (0.1/1/10 mg/kg) of various antibiotics belonging to the sulfonamides class to *Hermetia illucens* larvae. Results showed that the larvae contaminated with the highest dose had lower survival, pupation, and eclosion rates, but also lower body weight when compared to non-contaminated larvae. Moreover, contamination elongated larval stage duration and caused a later appearance of adults. Developmental issues and high mortality rates have also been observed in *Tenebrio molitor* [173,176], with varying severity depending on the dosage or the type of drug administered. In the literature, many sources focused on *Bombyx mori* rearing for its role in silk production, evaluating both positive and negative effects. Drugs and antibiotics testing on this species is often addressed to sericulture, and evaluations focus on silk production characteristics, such as cocoon and shell weight, shell ratio, filament length, and cocooning percentage [168,171,178,179,182,186,192,193,194]. These studies are not included in this review, as sericulture does not involve the use of *Bombyx mori* as a food or feed ingredient. As well as dosage, the way of administration of pharmaceuticals can also affect the intensity of the effects of such substances when applied to insects. The most common way of inclusion is by inoculation in the substrate, therefore, contamination by ingestion. In some cases, antibiotics and drugs were also applied by injection [172,173,176,195,196,197,198,199,200,201], which resulted in more severe effects. In 1973, Pansa et al. [195] tested different methods of administering the antibiotic valinomycin to adult house flies (*Musca domestica*) in order to evaluate the population’s LD_50_. They discovered that injection caused the highest mortality, with previous movement issues in individuals, followed by ingestion and topical application. In some cases, inclusion of pharmaceutical substances can also induce issues with the biosynthesis of components. Some anti-tumoral drugs (actinomycin D and mitomycin C) have been proven to interfere with biological pathways for the synthesis of RNA, nucleic acids, and proteins by inhibiting the incorporation of some nucleosides, such as in mealworms [172,173] and silkworm [196,201]. In some studies, researchers also focused on drug residues in insects following an administration period of variable duration [170,181,184,185,199,202,203,204]. This aspect is of critical importance in the context of edible insect farming, particularly when insects are intended for human or animal consumption. The presence of antibiotics or pharmaceutical residues raises concerns not only regarding consumer safety but also with respect to the growing issue of antimicrobial resistance. In the literature, insects have also been employed on spiked substrate in order to delineate degradation processes in substrates, to assess insects’ degradation capacity and explore their potential application in the breakdown and removal of contaminants from complex matrices [205,206,207,208]. The persistence of active substances in insects is highly dependent on the employed dosage. Gao et al. [181] discovered residues of one antibiotic (sulfadiazine) out of four tested in *Hermetia illucens* larvae, and only when medium and high dosages were used (1–10 mg/kg). In 2024, Hoek-van der Hil [185] analyzed various substrates to evaluate the natural presence of antibiotics and potential accumulation in black soldier fly larvae. Only oxytetracycline was discovered in a detectable amount when pig manure was employed as a substrate. Many studies found in the literature also investigate frass. Various insect species can act as active agents reducing the amount of drug residues in substrates, mainly manure [205,207,208]. This comprises a different target, hence not included in this review. Clearly, antibiotics and veterinary drugs do have beneficial effects if used correctly, but if misused or applied carelessly, they can become a double-edged sword. Other than the outcomes previously cited, active substances can bring a wide range of other detrimental effects on insect organisms, such as microbiota imbalances [171,186,197,209,210], decreased antioxidant activity [181,211,212], morphological abnormalities [196,199,200,213], and overall discomfort. The studies presented in this review, along with the negative effects observed following the improper use of pharmaceuticals in insects, highlight the critical importance of monitoring the presence of such contaminants in substrates intended for insect rearing. In the near future, attention should also be directed toward the development and administration of veterinary drugs specifically formulated for insects. Although rigorous substrate inspection is a time-consuming process in the context of insect farming, its absence—and the consequent presence of contaminants—can negatively affect insect survival and growth at various life stages, reproductive performance, general health, and overall welfare, ultimately resulting in significant economic losses if not adequately managed. Furthermore, the potential accumulation of contaminants in the insect body raises serious concerns in terms of food safety and may contribute to the growing global issue of antimicrobial resistance, which is already recognized as a major public health threat. Considering the rapid increase that edible insects farming is facing, the lack of regulation and guidelines on the use of veterinary drugs represents a major concern that needs immediate attention, especially if seen in an organic farming optic.

## 5. Pesticides

Under the term “pesticide” are included all those substances intended to protect plant organisms from any kind of harmful organism, or to prevent their attack. This includes insecticides, herbicides, fungicides, nematicides, and soil fumigants [218]. They are also considered biocides, as they can eradicate vector organisms—such as insects, rodents and other pests—that may transmit diseases to plants and crops [219,220]. A major concern regarding the use of these substances is their potential toxicity to non-target living organisms, including humans and animals [221]. Given the potential hazards they pose, these substances are subjected to some of the most stringent regulatory controls worldwide, both regarding their placing on the market (Reg. (EC) 1107/2009) [222] and the residue levels in food (Reg. (EC) 396/2005) [223]. Their pathways of degradation in the environment are multiple, and the degradation process itself is influenced by various factors, including intrinsic characteristics and properties of the substance, its concentration and microbial activity in the soil [218]. Nevertheless, residues are often found in the environment and, given the bioaccumulative potential that some organic compounds have, this holds a great risk in terms of safety issues in the food supply chain [224,225]. This clearly poses a risk in the detection of pesticide residues in crops, soil, water, but also in agricultural products, animal products and feed, and animal manure. Pesticides have a long list of negative side effects on human health—both acute and chronic—and on animal health [224]. Given their high toxicity, the European Union has issued regulations about maximum residue levels (MRLs) of pesticides in food and feed of plant and animal origin [223]. MRL is intended to be the highest level of pesticide residue that is legally tolerated in food and feed when good agricultural practices are applied. Currently, more than 1000 pesticides are included in the MRL legislation, and if a set substance is not included, there is a default general limit set at 0.01 mg/kg. Moreover, EFSA provides an annual report assessing pesticide residue levels in the European market, in order to assess the risk of finding non-compliant samples, which in 2022 were 2.2% [220]. In this regard, some producers choose to maintain a high level of sustainability by completely avoiding the use of synthetic and artificial pesticides altogether. This approach is adopted by organic farmers, who, as stated in Regulation (EU) 2018/848 [12], prioritize good agricultural practices and rely on the use of natural and organic inputs. The organic farming regulation allows the use of pesticides in exceptional circumstances, if authorized by Regulation (EC) 1107/2009 [222] and if consistent with organic farming principles, but the preference still goes to practices that do not comprise the use of synthetic substances. Insecticides are often non-specific, thus affecting all invertebrates present, including non-target organisms. Among the most impacted are pollinators. The contamination of crops with these active substances has a detrimental effect on these beneficial species as well [226,227]. Currently, with the advent of the edible insect sector, these production species are also at risk. In insect farms, as well as lab-scale farms, edible insects are reared mainly on vegetable substrates and given the high frequency of pesticides—including insecticides—found in food and feed products, this could represent a serious risk if the substances’ concentration is elevated, not only in terms of productive yields, but also in survivability rates. Moreover, in an organic farming optic, residues of pesticides in insect products (that may come from contaminated substrates) should not be present, in compliance with the organic regulation. Therefore, pesticide residues pose an issue both in terms of insect health but also from a legislative point of view. Numerous studies in the literature have assessed the impact of pesticides at MRLs, as well as at concentrations above or below these thresholds. To avoid the risk of using contaminated substrates—thus, impacting on insects’ rearing characteristics—the latter should be hazard-free, but in-depth checks on the presence of active substances can be extremely time-consuming, as well as financially demanding. Moreover, if insects were to bioaccumulate these toxic compounds, the presence of pesticides would be found in the final product, posing a risk for the consumer, whether human or animal. In Table 5, all the studies displayed analyze the effects of various types of pesticides in edible insect species. Several studies have investigated the bioaccumulation potential of specific compounds. According to the literature, *Hermetia illucens* larvae appear to effectively metabolize several pesticide residues present in the substrate, reducing the level of contaminants while showing no detectable nor significant residues in their bodies [228,229,230,231,232]. On the contrary, in some other cases, residues of pesticides or secondary metabolites could be detected in insects (mainly affected by time), such as in *Hermetia illucens* larvae [233,234], in *Tenebrio molitor* larvae [203,231], in *Alphitobius diaperinus* larvae [235,236] and in *Bombyx mori* larvae [237,238,239,240,241,242,243,244,245]. In the cited studies, different tissues have been analyzed in order to detect the accumulation of pesticides, which have been found in fat body tissue, hemolymph, eggs, and whole larvae. Moreover, considering the variability reported in the literature regarding the bioaccumulation of active substances, it can be concluded that it might depend on multiple factors, including the concentration of the substance, the duration of exposure, the species, and the age of the larvae. The preferred way of testing the effects of pesticides is to artificially inoculate the active substances by spiking substrates at known concentrations. Other ways of inclusion, less practiced, are injection and topical application (Table 5). The first symptoms of pesticide contamination in larvae appear to be issues in growth and development. Together with lower larval, pupal, and adult weights and lower survival rates, longer larval periods, issues in moulting, and lower adult emergence rates are often reported. From a production perspective, the deterioration of one or more of the aforementioned traits would lead to a rapid decline in both productive performance and, consequently, the economic viability of the farming system. Studies performed on *Hermetia illucens* show that a combination of dosage, period of administration and amount of administered substance can cause lower larval weights, lower survivability rates, and overall developmental issues [229,230,231,233,234,246]. This has also been demonstrated in *Tenebrio molitor* [231,247,248], *Alphitobius diaperinus* [235,236,248], *Acheta domesticus* [190], *Gryllodes sigillatus* [249], *Locusta migratoria* [250] and *Bombyx mori* [191,240,241,243,251,252,253,254,255,256,257,258,259,260,261,262,263,264,265,266,267,268,269,270,271,272,273,274,275,276,277,278,279,280,281,282,283,284,285,286,287,288,289,290,291,292]. In 2021, Meijer et al. [233] analyzed the effects of six insecticides belonging to different classes of compounds on the growth and survival of *Hermetia illucens* larvae to evaluate the detrimental effects of these substances. In the first experiment, they tested the substances by sprinkling the rearing substrates at the European MRL level. Based on the sorted effects, in a second experiment, they tested different concentrations, both higher and lower than the MRL. For some compounds (e.g., chlorpyrifos, propoxur, tebufenozide), the survival rates and larval weights of spiked larvae and control larvae did not show any statistical differences, meaning that the current set MRL is sufficiently low to avoid adverse consequences in larvae rearing. For some other active substances (cypermethrin and spinosad), instead, concentration at the MRL level was enough to reduce the survivability of larvae. This highlights that different molecules may exert distinct effects, follow diverse metabolic pathways, and elicit varied reactions in larval organisms, thus presenting different levels of risk and toxicity. Other factors, together with the administration of pesticides, can influence the effects of toxicity on survival rate and insect growth and development. For instance, rearing conditions: according to the literature, both *Gryllodes sigillatus* [249] and *Locusta migratoria* [293] showed higher susceptibility to pesticide contamination in gregarious conditions compared to solitary conditions. Another aspect that is affected by the presence of toxic molecules in insects is the reproductive capacity and fertility traits. Many insecticides are formulated in order to inhibit insect growth and reproductive capacity and aim at the eradication of pests in crops or stored grains. According to various studies, the presence of sub-lethal residues can still cause detrimental effects on non-target insects’ fecundity, leading to suppression of fertility, egg production, and survival. This has been shown in several insect species relevant to production, such as *Musca domestica* [294], *Tenebrio molitor* and *Alphitobius diaperinus* [248], *Acheta domesticus* [190,295], *Gryllodes sigillatus* [296], and *Bombyx mori* [240,244,255,283,297,298,299]. Pesticide contamination can also affect movement. This has been demonstrated in *Musca domestica* flies [300,301,302], in *Tenebrio molitor* larvae, pupae and adults [247], in *Alphitobius diaperinus* adults [132], and in *Locusta migratoria* [303] after topical application of different substances. In flies, the first reaction observed following the exposure is a highly increased activity, which results in convulsive flying, followed—in all cases—by tetany and almost complete depression of movements. In yellow mealworms, in all the tested life stages, the application of the pesticide caused immobility in 50% of the exposed insects, which was affected by life stage and time. Indeed, adults, which were the most susceptible, showed decreased larval immobility over time, while immobility of pupae increased with time, showing a delayed response. Similarly, several studies conducted on *Bombyx mori* larvae have reported a distinct set of symptoms associated with pesticide intoxication, including impairments in motor function, after ingestion of contaminated leaves [243,245,259,260,262,263,267,273,274,275,282,283,285,290,291,304,305,306,307]. These movement issues include paralysis, frantic and chaotic movements, head nystagmus, cramps and convulsions. Moreover, intoxication symptoms also comprise vomiting, softening of the body, and body shrinkage, clinical features that further highlight the toxicity of certain substances to these insects. Among the primary physiological issues reported in insects, oxidative stress has been frequently associated with pesticide contamination, with a consequential increase in reactive oxygen species (ROS) [191,238,283,288,308,309,310,311]. Oxidative stress can be defined as a condition in which the production of ROS exceeds the capacity of the antioxidant systems, resulting from an imbalance between the two [312]. Several studies on various edible insect species have reported that pesticide contamination leads to increased activity of antioxidant enzymes and a general induction of detoxification activities and immune defence pathways, enabling the insect body to counteract the toxic effects of these compounds [191,238,239,243,260,262,270,273,274,275,277,279,280,283,290,304,305,311,313,314,315,316,317,318,319,320,321,322,323,324,325]. A wide section of studies on pesticide effects is related to *Bombyx mori* farming. Silkworm larvae have long been of particular interest, due to their role in the production of silk. Since 2021, with the EU approval on the use of processed *Bombyx mori* proteins in animal feed [26], concerns regarding the effects of pesticides have extended to larvae reared for feed production as well. One of the main evaluated outcomes on *Bombyx mori* larvae after exposure to pesticides is the differential gene expression caused by the contamination with toxic substances, causing mutagenic effects at different levels. In many cases, researchers focused on the silkworm’s ability to produce silk under pesticide stress caused mainly by contaminated diets [241,245,252,253,259,265,269,270,273,275,286,289,316,326,327,328,329,330]. Secondarily, another major aspect that is compromised by toxic compounds is the midgut health, as well as the microbiota composition [191,258,261,263,267,270,271,272,277,283,286,318,323,331,332,333,334]. Other than that, mutagenic effects can also compromise the transcription of other genes involved in the expression of various characters [287,335,336,337,338,339,340]. Pesticides can exert many other detrimental effects, as they are intended for eradicating target organisms, which may negatively impact the performance of crops or farmed animals. In our bibliographic list are included studies analyzing effects on the endocrine system [237,240,260,286,341,342,343], respiration rates [344,345], nutrient metabolism, transportation and accumulation [345,346,347,348,349,350,351,352,353], and overall sensitivity to these active substances and their toxicity [354,355,356,357,358,359]. It can be stated that pesticide residues in substrates intended for edible insects may exert negative effects on insect health and productive traits, varying in both intensity and manifestation. For this reason, the concentration of such active substances should be kept as low as possible or, ideally, entirely absent. This would represent the optimal scenario, particularly in the context of a hypothetical organic edible insect sector, considering that current guidelines under Regulation (EU) 2018/848 [12] on organic production prohibit the use of synthetic chemical substances, including pesticides. Moreover, minimizing or eliminating pesticide residues would enhance the overall safety of the insect production chain—whether conventional or organic—thereby ensuring a higher-quality and safer end product from a food safety perspective.

## 6. Conclusions

Taking into account the factors examined in this review and in the current literature, the establishment of an organic edible insect sector, along with dedicated regulatory guidelines, appears feasible. Research on insect stress and welfare is essential not only for understanding their strengths and physiological adaptations to stressful environments, but also for optimizing rearing conditions, in order to improve both their productivity and overall well-being. Similarly, the use of chemically synthesized compounds, such as pharmaceuticals and pesticides, must be carefully regulated, as required by current organic regulations—particularly in light of the ways in which insects metabolize these substances. Nonetheless, several critical issues remain to be addressed or deepened, such as the housing and husbandry practices, the management of insect byproducts, or the conversion from conventional to organic insect production, in order to provide an overall comprehensive overview of this emerging sector.

## Figures and Tables

**Table 1:** Main environmental factors (temperature, humidity, light) as stressors and their effects on edible insects.

Order	Specie	Stress	Evaluation	Stage	Effects	Reference
Dip	*Hi*	RH	Eggs eclosion Adult emergence	E P	Low RH: >mortality, slower development (E, L and P), <A emergence, <A longevity	[125]
Dip	*Hi*	T	Survival rate Development Longevity Reproduction Body mass	E L PP P A	Low t°: >E eclosion time, >L and PP development 35 °C: <E eclosion time, >eclosion rate 30 °C: <PP development 10 °C and 42 °C: no E survived 40 °C: no PP High t°: <L survival rate, <P survival <A survival at extreme t°, >fecundity at 30 °C Higher weights at 35 °C (PP, P, A)	[80]
Dip	*Hi*	H	CCRT (Chill-Coma Recovery Time), HKDT (Heat KnockDown Time), CTmax (critical Thermal maximum) Movement and middle cross (MC)	A	Differences in time for movements, MC, total distances based on acclimation t°, species and t° Changes in CTmax based on acclimation t°	[111]
Dip	*Hi*	T	CTmax (critical Thermal maximum) and CTmin (critical Thermal minimum)	A	No significant effects of age, sex and size on CTmax >age, >CTmin (worse in females) <size, <CTmin >size, >CTmin (in females)	[110]
Dip	*Hi*	T	Growth rate Metabolism	L	>t°, <growth and metabolism	[82]
Dip	*Hi*	F L	Volatile organic compounds (VOCs)	L	Changes in VOCs emission (no statistical differences)	[130]
Dip	*Md*	G RH T	Calorespirometric investigations	P	>t°, <development	[124]
Dip	*Md*	D H	Locomotor activity	A	>D, <activity >t°, <activity	[118]
Dip	*Md*	H	Locomotor activity Flight performances Morphological measurements	A	Changes in survival rates, flight and locomotor activity based on population and sex	[77]
Dip	*Md*	H	CCRT (Chill-Coma Recovery Time), HKDT (Heat KnockDown Time), CTmax (critical T maximum) Movement and middle cross (MC)	A	Differences in time for movements, MC, total distances (based on acclimation t°, specie and t°) Changes in CTmax (based on acclimation t°)	[111]
Dip	*Md*	H (short)	Morphogenetic consequences	A	>size of wings and changes in shape	[121]
Col	*Adi*	C	Days of survival Recovery from chill coma	A	<t°, >time: <days of survival; <time of chill coma in males later entry in chill coma = no recovery = death	[87]
Col	*Adi*	F T	Metabolic responses	A	>survival at low t° during F Non-F beetles: <water content at 12–16 °C 12 °C: <triglycerides and glycogen in non-F beetles <triglycerides, glucose, glycogen and protein >glycerol in F beetles 16–20 °C: <triglycerides, glycogen, glycerol, glucose, protein in F beetles	[131]
Col	*Adi*	T	Days of survival	A	6 °C: >energy consumption <fresh mass (males) <water ratio (females) 10 °C: >size = >survival, <weight loss	[88]
Col	*Adi*	F T	Survival O_2_ consumption	A	Non-F beetles: >O_2_ consumption; >t°, >O_2_ consumption	[89]
Col	*Adi*	T F	Survival ratio Free amino acid levels	A	Longer survival for females >free amino acids with C	[84]
Col	*Adi*	T	Survival Reproductive capacity	A	>t°, <survival; >survival with acclimation >t°, >number of L; >L with acclimation	[112]
Col	*Adi*	T	Chill coma temperature O_2_ consumption	A	<chill coma t° in non acclimated insects; <acclimation t°, <O_2_ consumption	[75]
Col	*Adi*	T	Growth rate Nutritional content Respirometry Daily energy assimilation (DEA) Conversion efficiency (CE)	L	>t° (31 °C), >growth; <t° (15.2 °C), <growth minimum growth at 37–39 °C Max lipid at 37 °C—max protein at 15.2 °C VO_2_: >when fasting (except at 15 °C); >t°, >VO_2_ >DEA and CE at 31 °C	[85]
Col	*Adi*	Ch T	Recovery time Active Larvae	A	Recovery time based on population/duration of exposure to extreme t° <number of active L with insecticide <probability of recovery with insecticide	[132]
Col	*Tm*	T	Survival capacity	E L P A	>RH, >water uptake; no embryo development at <t° <E weight at <t°; <RH, higher E mortality >t° and >RH, >young L mortality	[72]
Col	*Tm*	RH T	Survival, thermal acclimation Water loss Metabolic rate	L P A	>resistance of pupal stage; >stress at <RH; >resistance of older L at <RH; no E development at <RH and <t°; no E laying at <t°;	[123]
Col	*Tm*	T	Critical Thermal limits (critical Thermal maximum CTmax and critical Thermal minimum CTmin)	A	<CTmax and CTmin with faster rates of change in t Changes in CTmin after 35 °C exposure Longer time of reacclimation to 25 °C after exposure to 15/35 °C	[109]
Col	*Tm*	T	Growth rate Nutritional content Respirometry Daily energy assimilation (DEA) Conversion efficiency (CE)	L	>t (31 °C), >growth minimum growth at 37–39 °C Max lipid at 31 °C—min protein at 31 °C VO_2_: >when F (except at 15 °C and 33 °C) >DEA at 31 °C	[85]
Col	*Tm*	RH	Survival and growth rate Length	L	>RH, >mass and length	[127]
Col	*Tm*	RH T (transport)	Hatch rate	E	<10 to >35 °C: <hatch rate >3 days at 5–10 °C: <hatch rate <RH at >t°: >hatch rate (if <6 days)	[126]
Col	*Tm*	H pathogen exposure	Survival Development and fitness measurements Immune response	L	>survival rates for L exposed to *M. brunneum* after short H; Temporary (5 d) <body weight gain in H (L) >antibacterial activity in haemolymph after HS; <haemocytes concentration after exposure to *M. brunneum* >body weight in larval O of females exposed to short H >haemocytes concentration after long H <haemocytes after long HS + exposure to *M. brunneum* >number of exuviae after H	[86]
Col	*Tm*	T	Recovery from chill coma Resistance to Heat shock	A	C tolerance test: better recovery in A reared at low t° better resistance to H shock in A reared at high t° <t° acclimation: >resistance to low t° >t acclimation: >resistance to high t°	[97]
Ort	*Lm*	H	Survival rate Heat Shock Proteins (HSPs)	A	>47 °C <survival rate >t°, new HSPs	[83]
Ort	*Lm*	H (shock)	Thermosensitivity of the flight system	A	>t°, <wingbeat frequency Acclimation: >chances of survival >thoracic t°, >frequency of flight rhythm	[120]
Ort	*Ado*	T	Chill coma recovery Locomotor activities Standard metabolic rate Mortality	A	33 °C acclimation: >chill coma recovery >t°, >running speed and jumping distance >t°, >standard metabolic rate >mortality in 33 °C and 25 °C acclimation	[105]
Ort	*Ado*	Ionizing R	Hormetic effects on adult reproduction and offspring performance	A O	Low dose: >female fecundity, >O size, >O performance <survival to reproductive maturity with high dose	[133]
Ort	*Ado*	C	Spermatophore production Spermatophore transmission	A	<t°: less likely to transport/transmit spermatophore	[114]
Ort	*Ado*	R	Growth rate	A O	<maturation mass, <growth rates	[134]
Ort	*Ado*	T (transport)	Survival	E N	<survival 3 days after transport at <t° (5 °C) <survival 6 day after transport at >t° (35 °C) No growth in transport at <t° (5 °C and 25 °C) >t°, earlier hatching of E after transport >t°, >hatching rate	[90]
Ort	*Ado*	F L	Haemocytes count Protein content Body weight	A	16 L:8 D, >haemocytes in males 12 L:12 D, >haemocytes in females <haemocytes in females with F 16 L:8 D, <protein content in haemolymph	[128]
Ort	*Ga*	T	Mortality Reproductive capacity	A	40 °C = 50% mortality in females 34 °C = decreased oviposition	[115]
Lep	*Bm*	H	Larval mortality Effective rate of rearing (ERR%)	L	100% mortality at 45 °C >mortality of young L with >t° >t°, >ERR%	[74]
Lep	*Bm*	H	Catalase (CAT) activity	L	>CAT activity in fat body (based on breeds) at 35 °C <CAT activity in fat body at 40 °C <CAT activity in midgut and haemolymph at <40 °C (based on breeds)	[122]
Lep	*Bm*	T	Larval mortality Egg hatchability Pupation rates	E L	t° < or >25 °C = worse biological performances RH < 70–80% = worse biological performances <RH and >t°: >mortality	[76]
Lep	*Bm*	T	Yield Rearing traits	L	Rearing traits: based on genotype/environment interactions	[78]
Lep	*Bm*	H	Reproductive capacity	A	>t° (42–45), <gonadosomatic index >t° (42), <fecundity 45 °C = death 39–42 °C, >vitellogenin mRNA expression 45 °C, <vitellogenin mRNA expression	[79]
Lep	*Bm*	F T	Thermal tolerance Chill Coma recovery time (CCRT)	L	F, <CCRT F + Heat shock, minimum CCTR	[106]
Lep	*Bm*	H	Heat shock proteins (HSPs)	L	Different HSPs (variations in t° and sex)	[96]
Lep	*Bm*	H	Body weight Mortality	L	>t°, <body weight	[81]

Dip: Diptera; Col: Coleoptera; Ort: Orthoptera; Lep: Lepidoptera; *Hi*: *Hermetia illucens*; *Md*: *Musca domestica*; *Adi*: *Alphitobius diaperinus*; *Tm*: *Tenebrio molitor*; *Lm*: *Locusta migratoria*; *Ado*: *Acheta domesticus*; *Ga*: *Grillus assimilis*; *Bm*: *Bombyx mori*; RH: relative humidity; T: thermal (stress); H: heat (stress); C: cold (stress); F: fasting; L: light (stress); G: gas (stress); D: density (stress); Ch: chemical (stress); R: radiation; E: eggs; L: larvae; N: nymphs; PP: prepupae; P: pupae; A: adults; O: offspring.

**Table 4:** Effects of various veterinary drugs on edible insects.

Order	Specie	Veterinary Drug	Inclusion	Dose	Stage	Evaluation	Effects	Bioaccumulation	Reference
Dip	*Hi*	Carbamazepine Roxithromycin Trimethoprim Azoxystrobin Propiconazole	Diet	Carbamazepine 1.8–1.9 mg/g Roxithromycin 5.8–5.9 mg/g Trimethoprim 5.9–9.9 mg/g Azoxystrobin 2.4–4.6 mg/g Propiconazole 3.2–14.1 mg/g	L	Larval growth Nutritional composition Residues	>fat content with contaminated substrates	Only trimethoprim	[170]
Dip	*Hi*	Moxidectin	Diet	645 ng/g (Moxidectin)	L	Larval growth Survival rates	<larval growth <survival rate with contaminated feed	Not evaluated	[180]
Dip	*Hi*	Sulfamonomethoxine Sulfamethoxazole Sulfamethazine Sulfadiazine	Diet	Low (0.1 mg/kg) Medium (1 mg/kg) High (10 mg/kg)	L P	Survival rate Body weight Duration of larval/pupal stage Pupation/eclosion dynamics Antioxidant activity (catalase CAT, peroxidase POD, superoxide dismutase SOD, total antioxidant capacity T-AOC Drug residues	<survival at high dose <pupation rate at high dose <eclosion rate at high dose >contamination, <body weight High dose, >larval stage duration Delay of the peak of pupation at high dose Later appearance of A at high dose >cumulated eclosion rate at low/medium dose <CAT activity at high dose >dose, <POD activity >dose, <SOD activity <activity of T-AOC at high dose	Only sulfadiazine was detected in medium and high dose treatments	[181]
Dip	*Hi*	Oxytetracycline	Diet	0/100/1000/2000 mg/kg	L	Degradation of antibiotic Effects on intestinal microorganisms	>degradation efficiency with larvae <growth rates and diet consumption levels (<3 d) >growth rates and diet consumption levels (>3 d) <gut microorganism diversity in the first 3 d >dose, >microorganism diversity overall Changes in gut bacterial community composition	Not detected	[206]
Dip	*Hi*	Oxytetracycline	In Oxytetracycline bacterial residue and soy	434.4 mg/kg 3042.3 mg/kg	L	Degradation of substances Weight gain Gut bacterial population	>dose, <degradation >dose, <weight gain >dose, <diversity of bacterial community	Not detected	[183]
Dip	*Hi*	Flubendazole (FLUB) Ivermectin (IVM) Doxycycline (DOX) Flumequine (FLUM) Sulfadiazine (SULF)	Diet	0.05 and 0.5 mg/kg (FLUB, IVM) 0.5 and 5 mg/kg (DOX, FLUM, SULF)	L	Survival rate Larval growth Drug residues Mass balance	<growth with IVM 0.5 mg/kg Mass balance: 40% of the drugs were found back	Larvae: DOX, FLUM, SULF, IVM, FLUB and FLUB metabolites	[184]
Dip	*Hi*	Lincomycin fermentation residues (LFR)	Diet	100 g LFR/100 g of what bran	L	Degradation	84.9% degradation rate >richness and diversity of larval microbiota >six lnu genes encoding nucleotidyl transferases	Not detected	[210]
Dip	*Hi*	Various	Diet	Naturally present in various substrates	L	Presence/Not presence	Not measured	Only oxytetracycline with pig manure substrates	[185]
Dip	*Hi*	Enrofloxacin Oxytetracycline Sulfamethoxazole Narasin Salinomycin Toltrazuril Eprinomectin	Diet	0.5 and 5 mg/kg (Enrofloxacin, Oxytetracycline, Sulfamethoxazole, Eprinomectin) 5 and 50 mg/kg (Narasin, Salinomycin, Toltrazuril)	L	Survival rate Larval growth Drug residues Molar mass balance	Eprinomectin-5: <survival rate Oxytetracycline-5, Eprinomectin-0.5/5: <larval growth	Not detected	[214]
Dip	*Md*	Amphotericin Mycoticin Flavofungin Filipin	Diet	Amphotericin 60 mg/flask Mycoticin 20 mg/flask Flavofungin 60 mg/flask Filipin 5/10/17 mg/flask	L A	Larval growth Mortality	Flavofungin: 100% mortality in 24 h Filipin low dose: slow larval growth Filipin medium-high dose: 100% mortality in 4 d Digitonin high dose: 100% mortality in 24 h Filipin: retarded E production and <% of flies that developed E and ovaries	Not evaluated	[174]
Dip	*Md*	Actinomycin D	Injection	Not reported	A	Amino acid incorporation by microsomal components	Inhibition of RNA synthesis No rapid incorporation of leucine in free ribosomes	Not evaluated	[201]
Dip	*Md*	Nystatin Amphotericin Tetrin Trichomycin Trichlorfon Filipin Candicidin Aureofungin	Diet	Nystatin 3.24/32.4 ppm Amphotericin 3.24/32.4 ppm Tetrin 32.4 ppm Trichomycin 32.4 ppm Trichlorfon 3.24 ppm Filipin 3.24/32.4/324 ppm Candicidin 32.4/324 ppm Aureofungin 32.4 ppm	A	Growth and development Mortality	Filipin: <growth, >mortality	Not evaluated	[188]
Dip	*Md*	Filipin	Diet	8/15/29 µmoles	L A	Mortality E production Effects of dietary cholesterol Loss of 32 P	>mortality, <E, >loss of 32 P With cholesterol: <mortality, >E, <loss of 32 P	Not evaluated	[175]
Dip	*Md*	Valinomycin	Diet Topic application Injection	1 mg/mL (mixed in sugar) 0.2 and 0.5 µL solution at different concentrations	A	Mortality (LD50)	Topic application: no toxicity Injection: >mortality, movement issues Consumption: >mortality, <ingestion	Not evaluated	[195]
Dip	*Md*	Racemomycin A, B, C Citromycin Streptothricin	Bait method	100/500/1000 ppm	A	Mortality rate	Different mortality rates (>than control) based on drugs and dosage	Not evaluated	[189]
Col	*Tm*	Actinomycin D	Injection in pupae	0.16 µg	P	Nucleic acid metabolism Protein biosynthesis	Issues with development from pupae to adults Inhibition of labelled uridine incorporation in RNA >incorporation of labelled glycine into protein 7 d after injection	Not evaluated	[172]
Col	*Tm*	Actinomycin D Mitomycin C Ouabain	Injection in pupae	Actinomycin D 3.2 µg/g live weight Mitomycin C 50 µg/g live weight Ouabain 7.3 µg/g live weight	P	Epidermal metamorphosis Nucleic acid synthesis	Antinomycin D: <RNA synthesis in epidermal cells, inhibition of eclosion, >mortality, abnormalities in cuticle development based on time of injection Mitomycin C: delay in attainment of peak thymidine uptake, differences in cuticle formation based on time of injection, inhibition of mitosis Ouabain: inhibition of cuticle formation based on time of injection, issues in ionic balance	Not evaluated	[173]
Col	*Tm*	Actinomycin D Mitomycin D	Injection in pupae	0.5/5 µg	P	Ecdysis	Antinomycin D: inhibition of the moulting process, narrowing of eyes with pigmented spots, delayed apolysis, >mortality Mitomycin D: developmental block (high dosage), limited apolysis, viscera with pupal form, failure in shedding old cuticle, irregular pigmentation of eyes, <number and size of caeca	Not evaluated	[176]
Col	*Tm*	Actinomycin D Mitomycin D	Injection in pupae	1–3 µL of 120 µM solution	P	Mortality Cuticle proteins	>mortality Modification of cuticula formation	Not evaluated	[177]
Col	*Tm*	Benalaxyl	Diet	50 mL solution	L	Enantioselective bioaccumulation	Different bioaccumulation based on the type of benalaxyl enantiomers	No significant accumulation in time	[202]
Col	*Tm*	Ampicillin Kanamycin	Diet Injected into the gut	Ampicillin 50/100 µg/mL Kanamycin 1/5/10/50 µg/mL	L	Bacterial Community Composition	Ampicillin: <bacterial communities diversity and size <in 16S rRNS gene copy number	Not evaluated	[197]
Col	*Tm*	Bentazone Clopyralid Fenpropimorph Linuron Mefenoxam Pendimethalin Pyrimethanil Tebuconazole	Diet	1000 mg/L	L	Bioaccumulation Presence of residues	Residues> than LOQ (except for bentazone and clopyralid) <residues after starvation	Detection of 6 substances out of 8	[203]
Col	*Tm*	Gentamicin Chloramphenicol Erythromycin Kanamycin sulphate Penicillin Tetracycline	Diet	30 µg/disc	L	Polyethylene degradation	Inhibition of gut microbes, but no inhibition of low-density polyethylene degradation	Not evaluated	[209]
Col	*Tm*	Acyclovir Amoxicillin Carbamazepine Chloramphenicol Chlorpromazine Cimetidine Cloxacillin Codeine Diazepam Diphenhydramine Furosemide Ketoprofen Mepivacaine Metoprolol Molnupiravir Nimesulide Paracetamol Salicylic acid Sulfadiazine Trimethoprim	Injection in adults	LD50 LD50/2	A	LD50/Toxicity	Death	Not evaluated	[198]
Col	*Tm*	Tetraconazole	Diet	2/20 mg/kg	L	Bioaccumulation	Bioaccumulation is higher in the first 3 d	Detected	[204]
Col	*Tm*	Irinotecan (IRI) 5-fluorouracil (5-FU)	Injection in larvae	IRI 10/75 mg/kg 5-FU 12.5/100 mg/kg	L	Health index Glucose level and ALT (Alanine aminotransferase) activity	Worse health index >mortality >dose, >number of cells darkening <glucose level <ALT activity Changes in the muscular layer >visceral surface area <external diameter and lumen diameter IRI: >lumen perimeter 5 FU: >external perimeter >histological score	Not evaluated	[187]
Ort	*Lm*	Actinomycin D (ACT) Cycloheximide (CLX)	Injection in nymphs	Actinomycin D 1/2/3/4 µg/g fresh weight Cycloheximide 100/200/300/400 µg/g fresh weight	L	RNA and protein synthesis inhibition	58% inhibition max (ACT) 4° instar: paler and greenish tint in 5° instar No ecdysis with injection late in instar CLX: max 90% inhibition Modified pronota and wing buds Different mortality based on the time of injection	Not evaluated	[213]
Ort	*Ado*	Miedzian 50	Spraying	0.1/0.01/0.001/0.0005/0.0001/0.00005/0.00001/0.000005/0.000001%	E	Mortality Development of eggs and new crickets	Accelerated hatching, <body size in first 2 months	Not evaluated	[190]
Lep	*Bm*	Aureomycin Chloromycetin	Oral administration	Not reported	L P	Larval growth	>9/10% in larval and pupal growth >8/14% in the production of silk	Not evaluated	[168]
Lep	*Bm*	Chloromycetin	Diet	20/30/40/50/60/70 mg/100 mL	L	Growth Production of silk	50–60 mg/kg live weight max beneficial effects Maximum effect in V instar Maximum effects with glycine	Not evaluated	[215]
Lep	*Bm*	Chloromycetin	Diet	200 mg/mL	L	Digestion and utilization of DM, N, mineral constituents, crude fat Composition of L Silk yield	<DM and fat digestibility >utilization of all constituents <utilization of nitrogen for silk production	Not evaluated	[192]
Lep	*Bm*	Chloromycetin	Aqueous form	250 µg/mL	L	O_2_ uptake Gut weight	>O_2_ uptake <gut weight >growth rate >glucose utilization in the guts	Not evaluated	[169]
Lep	*Bm*	Racemomycin D	Injection	50/100/150/200 µg/g	L	Mortality Body weight Excreta Distribution of the drug	<movement after 48 h Change in skin colour to yellow-brown 72 h: black skin <ingestion (100/150/200) Vomit Before 24 h: >weight After 24 h: <weight <excreta with time	Presence of compound in blood after 96 h No detection in feces after 24 h >residues in Malpighian tubes	[199]
Lep	*Bm*	Racemomycin D	Injection	150 µg/g	L	Excretion function	Abnormality of Malpighian tubes >uric acid in haemolymph >excretion of uric acid	Not evaluated	[200]
Lep	*Bm*	Mitomycin C	Injection	2.5/5/10 µg/g	L A	Wing formation Tritiated thymidine incorporation	Inhibition of wing formation based on dose and time of injection <Tritiated thymidine incorporation	Not evaluated	[196]
Lep	*Bm*	Cloxacillin Streptomycin Gentamycin Terramycin	Diet	0.05/0.1% in distilled water	L	Lipid and water content	>dose, <water content >dose, >lipid content	Not evaluated	[216]
Lep	*Bm*	Norfloxacin	Diet	50/100 ppm	L P	Feed conversion efficiency (Efficiency of Conversion of Ingested food index, ECI)	>digestibility of food source <excreta >ECI >conversion of food into a cocoon shell >larval biomass production	Not evaluated	[193]
Lep	*Bm*	Oflaxacin Acyclovir Griesovin	Diet	0.04/0.08/0.12%	L	Incidence of diseases	<incidence of disease and minimum mortality rate with 0.12%	Not evaluated	[217]
Lep	*Bm*	Iminoctadine	Liquid formulation	0.03–3% range	L	Moulting	>dose, deficient moulting	Not evaluated	[178]
Lep	*Bm*	Iminoctadine triacetate	Diet	0.03–0.1%	L	Development and mortality	Mortality (0.1%) 0.03%: longer larval period <intake	Not evaluated	[179]
Lep	*Bm*	Tebuconazole	Diet	0.4/2/20 mg/L	L	Silk parameters Juvenile hormone (JH1) and ecdysteroid (ES) levels	<cocoon weight, cocoon shell weight, cocoon shell rate Damage to the silk gland Downregulation of gene transcription involved in protein synthesis in the silk gland Decrease in mRNA expression <ES and >JH1	Not evaluated	[194]
Lep	*Bm*	Metformin	Diet	5 µL 0.1 M	L P	Lifespan Body weight Silk ratio Fecundity	>lifespan for M <cocoon-shell ratio <fecundity >lifespan in food stress	Not evaluated	[182]
Lep	*Bm*	Chlorampenicol Vancomycin	Diet	0.1 mg/kg	L	Growth indices Feeding behaviour Bacterial count Antioxidant enzyme activity Intestinal morphological and microbiota alterations Intestinal metabolic pathway	Promoted feeding behaviour <intestinal cultivable bacterial counts <antioxidant activity of some enzymes Oxidative damage to the intestine Perturbation of antimicrobial peptides gene expression Disintegration of some epithelial cells Variations in taxonomic composition of intestinal microbiota Variation in some metabolic pathways	Not evaluated	[211]
Lep	*Bm*	Florfenicol	Diet	0.06 g/L 0.12 g/L 1.2 g/L	L	Midgut physiology function Microbiota	High dose: <body/cocoon weight >conditional pathogens and <*Pseudomonas* and *Pedobacter* in microbiota >intestinal reactive O_2_ specie Loose muscle layer and gut goble cells atrophy >Lactobacillus, <intestinal fluid pH, <a-amylase and protease activities, >lipase activity	Not evaluated	[186]
Lep	*Bm*	Methyl-Thiophanate	Diet	2.5, 5, and 10 mg/mL	L	Toxic effect on physiological and transcriptomic analysis Oxidative stress (reactive oxygen species ROS, superoxide dismutase SOD, peroxidase POD, catalase CAT)	<weight autophagy in the midgut oxidative stress: activation of ROS, SOD, CAT, POD Differently expressed genes related to antioxidant defence, detoxification processes, lysosome biogenesis, and metabolic pathways	Not evaluated	[191]
Lep	*Bm*	Florfenicol	Diet	10 µg/L (LD) 50 µg/L (MD) 250 µg/L (HD)	L P	Cocoon production Fitness Development Midgut bacterial community Metabolome mRNA profile in midgut and fat Expressed genes in the midgut and fat	>whole cocoon weight and pupal weight at HD <cocooning rate and pupation rate Shift in microbial diversity and population Altered function in the midgut of microbial populations Alteration of metabolites for various biological pathways Differences in expressed genes	Not evaluated	[171]
Lep	*Bm*	Florfenicol	Diet	1.2 g/L	L	Antioxidant activities Mitochondrial damage	<antioxidant activity Cellular damage <phosphorylation pathway in the midgut >release of mitochondrial BmCytochrome c	Not evaluated	[212]

Dip: Diptera; Col: Coleoptera; Ort: Orthoptera; Lep: Lepidoptera; *Hi*: *Hermetia illucens*; *Md*: *Musca domestica*; *Tm*: *Tenebrio molitor*; *Lm*: *Locusta migratoria*; *Ado*: *Acheta domesticus*; *Bm*: *Bombyx mori*; E: eggs; L: larvae; P: pupae; A: adults.

**Table 5:** Effects of pesticides on edible insects.

Order	Specie	Substance	Dosage	Inclusion	Stage	Evaluations	Effects	Bioaccumulation	Reference
Dip	*Hi*	Cyromazine (CYR) Pyriproxifen (PYR) γ-cyhalothrin (CYH) Permethrin (PER)	CYR: 9 doses (0.0585–1.5 ppm) PRY: 14 doses 0.0316–1857 ppm CYH: 9 doses (0.061–1.5625 µg/cm^3^) PER:11 doses (0.65–37.5 µg/cm^3^)	Diet Treated paper	L A	Larval development Mortality Weight LC50	Cyromazine: 100% mortality with 1.5 ppm <prepupal weight <emergence rate Pyriproxifen: >prepupal weight with 58 ppm, <weight with 1857 ppm <% of L in prepupal stage LD50: 0.314–0.320 g/cm^2^ (CYH), 6.46–8.43 g/cm^2^ (PER)	Not evaluated	[360]
Dip	*Hi*	Chlorpyrifos Chlorpyrifos-methyl Pirimiphos-methyl	2.5 mg/kg of substrate	Diet	L	Larval survival Larval growth Concentration and bioaccumulation	No significant effects	Not detected	[228]
Dip	*Hi*	Chlorpyrifos Propoxur Cypermethrin Imidacloprid Spinosad Tebufenozide Piperonyl butoxide PBO	1° assay: MRLs 2° assay: >/<MRLs based on effects in the 1° exp	Diet	L	Larval survival Larval growth Concentration and bioaccumulation	1° exp: <larval survival with spinosad, cypermethrin and cypermethrin + PBO >larval biomass with imidacloprid <larval growth with spinosad, cypermethrin and cypermethrin + PBO	Exp 1: spinosad, cypermethrin, PBO Exp 2: all but propoxur	[233]
Dip	*Hi*	Malathion	0.005, 0.01, 0.015, or 0.02 mg/mL	Diet	L A	Concentration of reactive oxygen species ROS Oxidative damage Enzymatic antioxidant response non-enzymatic antioxidant response	<content of hydrogen peroxide and superoxide anion radicals mean values >mean protein carbonyls except for 0.02 mg/mL Max lipid peroxide concentration at 0.015 mg/mL, min at 0.2 mg/mL >Superoxide dismutase and catalase antioxidant activity at 0.02 mg/mL <Polyphenol oxidase activity at 0.2 mg/mL α, α-diphenyl-β-picrylhydrazyl (DPPH) >in 0.005 and 0.02 mg/mL >DPPH in control adult >Reduced glutathione in L and 0.01 and 0.02 mg/mL	Not evaluated	[311]
Dip	*Hi*	Lufenuron	0.069/0.149/0.282/0.878 mg/kg	Diet	L P A	Larval/pupal survival Morphology	<survival Morphological modification in L, P, A Presence of necrosis (at elevated concentration) Incomplete ecdysis in P	Not evaluated	[246]
Dip	*Hi*	λ-cyhalothrin	0.5, 1, 3, 5 mg/L in 25 mL solution	Diet	L	Larval survival Larval growth Concentration and bioaccumulation	100% mortality with 5 mg after 48 h 20% mortality with 3 mg <growth at 1 and 3 mg	Not detected	[229]
Dip	*Hi*	Cypermethrin (CYP) Deltamethrin (DEL) Permethrin (PER) Pirimiphos-methyl (PM) Chlorpyrifos-methyl (CM) Malathion (MAL) Piperonyl butoxide (PBO)	CYP: 0.5/1/1.6/2/4.5/8 mg/kg PBO: 40–40.3 mg/kg DEL and PER: 0.5–1.6 mg/kg PM: 2.5/3.4/5/7.5/10/10.1 mg/kg CM/MAL: 3.4–10.1 mg/kg + added with PBO + combined substances	Diet	L	Doses needed to affect larval growth	CYP MRL: insufficient to ensure optimal yields PM: >mortality, <yield DEL: >toxicity than CYP and PER Addition of PBO: <yield CM: <yield PM and MAL at a lot concentration: no effects on yield	No bioaccumulation for any treatment	[230]
Dip	*Hi*	Pirimiphos-methyl (PM)	10/20/40 mg/kg	Diet	L	Larval survival Larval biomass and weight Detection of metabolites Genotoxicity Mass balance Bioaccumulation	PM20: >50% survivability PM40: 10% survivability <body weight PM20, <at PM40 >body weight at PM10 6 metabolites detected in diet, 1 in L 98% of PM10 and PM20 metabolized	Not detected	[231]
Dip	*Hi*	Cypermethrin (CYP) Deltamethrin (DEL) Piperonyl butoxide (PBO)	CYP: 1.0–2.0 mg/kg DEL: 0.06/0.13/0.25/0.5/1.0 mg/kg CYP + PBO: 1/10 and 2/20 mg/kg DEL + PBO: 0.13/0.25 and 0.25/2.5 and 0.5/5 mg/kg	Diet	L	Toxicity, transfer and metabolization	>dose, <yield Concentrations >than maximum residue levels (MRLs) with higher exposure Overall recovery around 50%	Detection >MRL with higher concentrations	[234]
Dip	*Hi*	λ-chyalothrin Cypermthrin Acetamiprid Chlorpyrifos-ethyl Avermectin	Naturally present in biowaste streams	Naturally present in biowaste streams	L	Detection of pesticide residues	No detection in L	Not detected	[232]
Dip	*Md*	DDT	10 µg/mL	Topic application	A	Locomotion and activity	Initial irritation and hyperactivity, then activity decreased to 0 (based on dose)	Not evaluated	[300]
Dip	*Md*	DDT	1 µg/mL	Topic application	A	Flight and activity	Various effects based on pesticide: >interference of potentials of flight muscles, >motor activity, tetany, depression in activity, convulsive flight	Not evaluated	[301]
Dip	*Md*	Monitor Lindane Carbofuran	1 µL in acetone solution	Topic application	A	Thoracic temperature Flight Heart rate	>thoracic temperature Convulsions and convulsive flight >heart rate Tetany Excessive cleaning motions (varying based on substance)	Not evaluated	[302]
Dip	*Md*	Parathion Parathion methyl Malathion BAAY 22190 Chlorfenvinphos Tetrachlorvinphos Coumithioate G-23611 Carbaryl SD 8530 HEOD gamma-BHC p,p′-DDT Pyrethrum Resmethrin Tetramethrin	1 mg solution in water	Topic application	L A	Water loss Respiration	>loss of water by regurgitation or excretion, and transpiration <respiration rate	Not evaluated	[344]
Dip	*Md*	Benzoylureas	Various	Diet Injection	E L	Effects on E and L	Ovicidal activity Larvicidal activity	Not evaluated	[294]
Col	*Adi*	Cyfluthrin	5/10/20 mg/m^2^	In Petri dishes	A	Sensitivity to I and effects of temperature	Mobility impairment when exposed to cyfluthrin and heat spikes	Not evaluated	[132]
Col	*Adi*	Chlorpyrifos Propoxur Cypermethrin Imidacloprid Spinosad Tebufenozide Fipronil Pirimiphos methyl Piperonyl butoxide PBO	Chlorpyrifos: 0.05 mg/kg Propoxur: 0.05 mg/kg Cypermethrin: 0.3 mg/kg Imidacloprid: 0.1 mg/kg Spinosad: 2 mg/kg Tebufenozide 0.05 mg/kg Fipronil: 0.005 mg/kg Pirimiphos methyl: 0.5 mg/kg Piperonyl butoxide PBO: 6 mg/kg Cypermethrin + PBO: 0.3 + 0.6 mg/kg	Diet	L	Larval survival Larval growth Concentration and bioaccumulation	<total yield in Imidacloprid and Spinosad	Detection < LOQ, except for Spinosad	[235]
Col	*Adi*	Phospine	Bioassay 1: 50/100 ppm for 3 d Bioassay 2: 3000 ppm Bipassay 3: 3000 ppm for 90 m	Fumigation	E L P A	Mortality LT99 LT50	B1: 100% mortality after 7 d (A) and 14 d(L) (3 d of exposure 50/100 ppm) E: 5% survival after 7 and 14 d with 50 ppm B2: LT99 5.5, LT50 2.4 (L) Larval mortality: 20% (7 d), 40% (14 d) B3: mortality at 7 d 48.8%, at 14 d 53.3%	Not evaluated	[248]
Col	*Adi*	Spinosad SPI Imidacloprid IMI	MRLs and 10% of MRLs (2.0 and 0.2 mg/kg spinosad) (0.1 and 0.01 mg/kg imidacloprid)	Diet	L P A	Weight N. of L Pupation Eclosion Substance transfer	L: (parental generation P1 and offspring F1) SPI2: <weights in P1 and F1 IMI0.1: <weights in P1 SPI0.2: >number of L in F1 A: <weights in IMI0.1 >weights in SPI2 >pupation in P1 Transfer <10%	Transfer from feed to L of P1 and F1 < 10%	[236]
Col	*Tm*	2,4 D Bifenthrin Diflufenican Isoproturon	1000 mg/L	Diet	L	Bioaccumulation Presence of residues	Residues >than LOQ (except for 2,4-D and bifenthrin) <residues after starvation	Detection of 2 substances out of 4	[203]
Col	*Tm*	α-cypermethrin	31/250/750 ng/individual	Spraying	L P A	Locomotion Pupal development Toxico-kinetics Survival Enzymatic activities	Immobility after application >degree of deformities in beetles emerging from exposed P Internal concentration based on time and life stage <40% mortality, except for high dose in A, 90% mortality Different enzyme activity based on dose and life stage	Not evaluated	[247]
Col	*Tm*	Phosphine	Bioassay 1: 50/100 ppm for 3 d Bioassay 2: 3000 ppm Bipassay 3: 3000 ppm for 90 m	Fumigation	E L P A	Mortality LT99 LT50	B1: 100% mortality after 7 d (A) and 14 d(L) (3 d of exposure 50/100 ppm) E: 91.6 and 88.8% after 7 and 14 d with 50 ppm B2: LT99 4.34, LT50 1.78 Mortality: 60% (7 d), 93% (14 d) B3: 100% mortality	Not evaluated	[248]
Col	*Tm*	Pirimiphos-methyl (PM)	10/20/40 mg/kg	Diet	L	Larval survival Larval biomass and weight Detection of metabolites Genotoxicity Mass balance Bioaccumulation	PM20: >70% survivability PM40: 2% survivability >dose, <body weight 6 metabolites detected in diet, 1 in L PM10: >metabolization overall < 60% metabolization	Not detected	[231]
Ort	*Lm*	DDT Dieldrin Chlorfenvinphos Bioresmethrin	20 µL solution	Injection	A	Activity of hyperlipaemic hormone	>release of hyperlipaemic hormone from corpora cardiaca	Not evaluated	[341]
Ort	*Lm*	Bioresmethrin	0.01/0.1 µmol	Injection	A	Electrical activity of corpora cardiaca Hyperlipaemic hormone	Burst of electrical activity, >with >dose >release of hyperlipaemic hormone	Not evaluated	[343]
Ort	*Lm*	Lindane	3 µg/g in acetonic solution	Topic application	L	N-acetyldopamine (NADA) and N-acetyl5-hydroxytryptamine (NA % HT) in brain	<NADA and <NA 5 HT Initial hyperactive phase, later issues in movements, + paralysis >dopamine and 5-hydroxytryptamine	Not evaluated	[303]
Ort	*Lm*	Dieldrin Methomyl Fenitrothion Permethrin Tefubenzuron	Various based on LD50	Topic application	N A	Toxicity and resistance	>resistance with >in developmental stage for pesticides (except A) >resistance of younger L for growth inhibitor Tefubenzuron (A not affected) >resistance of solitary insects compared to gregarious ones	Not evaluated	[293]
Ort	*Lm*	Deltamethrin	0.2/0.23 µg/g	Injection	A	Kinetics of trehalosemia Glycogen and glycogen phosphorilase activity Biological activity of hemolymphatic peptide extracts	>trehalosemia in 24 h Retarded decrease in flight muscle glycogen Rapid decrease in fat body glycogen >activity of fat body glycogen	Not evaluated	[346]
Ort	*Lm*	Deltamethrin	LD 15, 25, 50, 75 and non-lethal	Injection	A	Glucose catabolic pathways	Changes in glucose catabolism when removing fat body based on dose and time of removal	Not evaluated	[347]
Ort	*Lm*	Deltamethrin Malathion Carbaryl	LD10, LD30 and LD50	Topic application	N	Analysis of cytochrome P450-like genes	Deltamethrin: significant effect on the cytochrome P450 enzyme activity >transcriptions of several cytochrome P450 genes	Not evaluated	[315]
Ort	*Lm*	Imidacloprid	0.1 µg–10 mg/mL solution with acetone	Injection	A	LD50 Behavioural responses Descending Contralateral Movement Detector Response (DCMD)	>doses, >movement issues, paralysis >dose, >mortality >resistance of F LD50 M: 2500 ng/g, LD50 F: 10,000 ng/g No reaction to visible stimuli <bursting	Not evaluated	[250]
Ort	*Lm*	Malathion Chlorpyrifos Deltamethrin Carbaryl	Malathion 0.48 μg/N Chlorpyrifos 0.18 μg/N Deltamethrin 0.015 μg/N Carbaryl 0.195 μg/N	Topic application	N	Susceptibility based on changes in cuticular structure	dsLmKnk3–50 = <chitin pesticides = >susceptibility	Not evaluated	[359]
Ort	*Ado*	Fenthion	300 mg/L	Topic application	E	Development and eclosion Mortality Cholinesterase (ChE) acitivty	>mortality of E depressed ChE activity at 8–9 d	Not evaluated	[295]
Ort	*Ado*	Karbatox 75 Lindane Gesagard 50	Karbatox 50: 0.1/0.01/0.005/0.001/0.0005/0.0001/0.00005/0.00001/0.000005/0.000001% Lindane: 0.1/0.01/0.005/0.001/0.0005/0.0001/0.00001/0.000005/0.000001% Gesagard 50: 0.1/0.01/0.001/0.0001%	Spraying	E	Mortality Development of E and new crickets	Karbatox 75 and Lindan (based on dosage): >mortality rates, presence of abnormalites in E and hatched crickets Gesagard 50 sorted no effects	Not evaluated	[190]
Ort	*Ga*	Glyphosate based herbicide (GBH)	0.864 mg	In water	N	Antioxidant enzymes Cholinergic enzymes Lipid peroxidation activity	>catalase activity in nymphal phase >cellular detoxification >efficiency of control of lipid peroxidation	Not evaluated	[325]
Ort	*Gs*	Chlordane Malathion Fenitrothion Pyrethrum Rotenone Sevin	0.005% solution in acetone	Topic application	A	Toxicity of substances based on rearing conditions	Crickets reared in crowded conditions > susceptible than solitary crickets 100% mortality in all conditions for chlordane chlordane > malathion > fenitrothion > pyrethrum > rotenone > Sevin	Not evaluated	[249]
Ort	*Gs*	Cythion	0.001–0.003% in water	Topic application	A	E production Fertility	<E production <hatching <E weight <weight of newly hatched N	Not evaluated	[296]
Lep	*Bm*	EDB (1,2-dibromoethane)	Acute toxicity: 10 µL acetone solution of EDB (5%) Chronic toxicity: 1000 ppm EDB emulsion	Topic application (acute) Diet (chronic) Vaporized	L	Development Weight Silk parameters Effects on next generation Mutagenic effects	Vapour effect: uneven development 100% mortality with 10.4 ppm exposure 100% mortality for newly ecdysis L with 41.6 ppm No pupation at 20.8 ppm	Not evaluated	[251]
Lep	*Bm*	MEP, MPP, EDB, PAP	Not reported	Leaves harvested from treated stumps	L	Residues in L	No residues < cocoon weight and shell weight	Not detected	[252]
Lep	*Bm*	Parathion Disulfoton Malathion	4–5 grades dilution from LD50-LD0	Diet Injection	L P	Reproduction	<number of E laid >% of abnormalities in E >E mortality Late hatching Retention of substances in pupal body Transfer from mother to E Respiratory inhibition <cholinesterase activity	Detected	[244]
Lep	*Bm*	1-citronellyl-5-phenylimidazole	3.12/6.25/12.5/25/50/100 ppm	Diet	L	Metamorphosis Pupation Ecdysis, an emergence, cocoon spinning	Precocious pupation (6.25–100 ppm) Earlier ecdysis and emergence <larval, cocoon and cocoon shell weights	Not evaluated	[253]
Lep	*Bm*	Azardirachtin	1/2 µg/g BW	Injection	L	Endocrine events	<weight and <pupation rate Morphological deformities No significant effects on endocrine events	Not evaluated	[254]
Lep	*Bm*	17 1,5 Disubstituted Imidazoles	1/4 µL/larva	Topic application	L	Development Toxicity	Precocious pupation with a variety of 1,5-disubstituted Imidazole No toxicity of the imidazole compound Toxicity of benzyl analogue	Not evaluated	[354]
Lep	*Bm*	Hexachlorocyclohexane	0.7 µg/larva	Topic application	E L P A O	Growth Cocoon production	Longer larval period <growth <fibroin content <cocoon weight and cocoon shell ratio <survival rate <number of E, <emergence of moths < reliability of cocoons Loss of body water Negative effects on offspring	Not evaluated	[255]
Lep	*Bm*	Fenoxycarb	100 pg/larva and 1 ng/larva	Diet	L	Juvenile Hormone Titer	Fenoxycarb: not a JH-esterase inhibitor Inhibition of larval prothoracic glands (PG) activity without modification of JH	Not evaluated	[342]
Lep	*Bm*	Dimehypo	0.0612/0.108/0.217/0.346/0.684/1.20 mg per leaf (acute toxicity) 1.7 × 10^−6^, 1.7 × 10^−5^, 3.4 × 10^−5^, 1.7 × 10^−4^ mg/day (spring); 1.7 × 10^−8^, 1.7 × 10^−7^, 1.7 × 10^−6^, 1.7 × 10^−5^ mg/day (autumn) (chronic toxicity)	Diet	L	Growth and cocooning	Acute toxicity: >mortality, <cocooning rate <wet weight of cocoons and cocoon layers Chronic toxicity: longer larval stadium and ecdysis <mean wet weight <cocooning rate (spring-autumn) Changes in the biosynthesis of fibroin and in the physiological activity of the posterior silk gland cell	Not evaluated	[289]
Lep	*Bm*	Fenitrothion Ethion	Lethal and sublethal dose	Diet	L	Carbohydrate metabolism Fat body	<pyruvate level and lactate dehydrogenase activity with >lactate levels <respiration rate at the tissue level (<enzymes of Krebs cycle)	Not evaluated	[345]
Lep	*Bm*	Fenoxycarb	1–2 µL solution	Topic application Orally administered	L	Leucine Uptake Lipid Composition of Midgut Brush Border Membrane	Feeding inhibition Frass inhibition >K^+^ dependent leucine uptake and accumulation in midgut <unidentified fatty acid	Not evaluated	[348]
Lep	*Bm*	Flufenoxuron	10–500 ppm	Diet	L	Nucleopolyhedrovirus infection	Enhanced viral infection >sensitivity to infection Fragile peritrophic membrane	Not evaluated	[355]
Lep	*Bm*	Flufenoxuron Chlorfluazuron Teflubenzuron Diflubenzuron Buprofezin	0.10%	Diet	L	Peroral infection by nucleopolyhedrovirus	Flufenoxuron: promoted BmNPV budded particle peroral infection Chlorfluazurn/teflubenzuron/diflubenzuron contaminated L did not pupate	Not evaluated	[256]
Lep	*Bm*	Fenoxycarb	0.2 µg/µL solution	Topic application	L	General and juvenile hormone esterase activity	>haemolymph GE and JHE activities Dauer L	In haemolymph and feces	[237]
Lep	*Bm*	Fenitrothion Ethion	LD50: 0.306 (F) 0.037 ppm sublethal dose: 0.061 ppm (F) 0.007 ppm (E)	Diet	L	Glycogen metabolism in haemolymph and fat body	<glycogen level in fat body >trehalose and glucose reserves in haemolymph and <in fat body >Glycogen phosphorylase and trehalase in the fat body <Food intake and feces defecated at lethal dose	Not evaluated	[349]
Lep	*Bm*	Rac- and S-metolachlor	600 mg/L in alcohol/water	Diet	L	Toxicity Enzyme activity	<haemolymph lactate dehydrogenase/catalase activity levels with rac <ACP activity with rac <midgut alkaline phosphatase activity with rac	Not evaluated	[350]
Lep	*Bm*	Clodinafop-propargyl	30, 60, 120, 240, 480 mg/L	Diet	L	Genotoxicity evaluation	>dose, >DNA damage <% of cocooning and pupating	Not evaluated	[257]
Lep	*Bm*	Organophosphorus and Pyrethroid I	2 µg/mL	Diet	L	Toxicity LC50	>toxicity LC50: from 0.06 to 4.11 mg litre-1 different levels of toxicity based on mixtures of different types of compounds	Not evaluated	[356]
Lep	*Bm*	Glyphosate Permethrin	10^3^ ppm	Injection	L	Effects on Glutathione S-Transferase Activity and gene expression	>mRNA level of BmGSTS2 >expression of the BmGSTS2 gene in midgut	Not evaluated	[313]
Lep	*Bm*	Diazinon	1 µg/g larva	Topic application	L	Antioxidant reactions	Induction of mRNA encoding manganese-containing superoxide dismutase (SOD), and omega-class glutathione S-transferases (GST) >ROS, <glutathione in fat body	Metabolites in the haemolymph	[238]
Lep	*Bm*	Fenoxycarb	1 ng/10 µL and 10 ng/10 µl	Topic application	L	Larval/pupal development Morphology of the midgut	>length of the last larval stage Inhibition of larval-pupal differentiation (spinning, gut purge and remodelling of midgut) Larval midgut degeneration and apoptosis >level of caspase 3	Not evaluated	[258]
Lep	*Bm*	Phoxim	4 µg/mL	Diet	L	Transcriptional characteristics of acetylcholinesterase genes	First >then <transcription levels of Bm-AChE-1 and Bm-AChE-2 in brain, fat body and silk gland First <then >transcription of Bm-AChE-1 and Bm-AChE-2 in midgut >dose, >mortality	Not evaluated	[314]
Lep	*Bm*	Avermectin	1.0/2.0/4.0 µg/L	Diet	L	DNA damage and gene expression in haemocytes	DNA damage of haemocytes Differential gene expression (<Polyubiquitin, ATP translocase, Glycoprotein, >ribosomal protein S18)	Not evaluated	[335]
Lep	*Bm*	Phoxim	4 µg/mL	Diet	L	Gene expression analysis	247 differentially transcribed genes Up-regulated genes: involved in glycometabolism, fat metabolism, protein hydrolysis electron transfer process Down-regulated genes: involved in cell division, transcriptional regulation, and intracellular signalling cascade	Not evaluated	[351]
Lep	*Bm*	Phoxim	4 μg/mL	Diet	L	Protein and Carbohydrate Metabolism	Beneficial effects of cerium with phoxim toxicity: >contents of protein, glucose and pyruvate, and carbohydrate metabolism-related enzyme activities by adding cerium chloride, <free amino acids, urea, uric acid and lactate levels and inhibited the protein metabolism-related enzyme activities	Not evaluated	[352]
Lep	*Bm*	Cypermethrin Dichlorvos Dimethoate Imidacloprid Monosultap Phoxim	2 mL solution	Diet	L	Toxicity test	Different toxicity based on pesticides	Not evaluated	[357]
Lep	*Bm*	Phoxim	4 µg/mL	Diet	L	Gene expression	Different gene expression on detoxification, immune response, stress response, energy metabolism and transport Upregulation of genes involved in detoxification	Not evaluated	[305]
Lep	*Bm*	Phoxim	4 mg/mL	Diet	L	Silk gland damage and cocooning + effects of TiO_2_ NPs	Severe damage to the silk gland structure <cocooning <expressions of silk protein synthesis-related genes Beneficial effects of TiO_2_ particles	Not evaluated	[326]
Lep	*Bm*	Phoxim	4 µg/mL	Diet	L	Gene transcription in the silk gland	270 differentially expressed genes related to apoptosis, detoxification and protein degradation Inhibition of fibroin synthesis	Not evaluated	[316]
Lep	*Bm*	Imidacloprid	2 mg/L	Diet	L	Microbial degradation Toxicological analysis	>antioxidant enzyme activities (SOD and CAT) in the midgut Haemocytes damages >lipid peroxidation and protein oxidation	Not evaluated	[317]
Lep	*Bm*	Phoxim	4 µg/mL	Diet	L	Midgut damages Antioxidant activities	<survival rate >malondialdehyde (MDA), carbonyl and 8-OHdG levels >ROS accumulation <activities of AChE, superoxide dismutase, ascorbate peroxidase, glutathione reductase, glutathione-S-transferase in midgut <levels of ascorbic acid, glutathione, thiol in midgut Beneficial effects of TiO_2_ NPs	Not evaluated	[288]
Lep	*Bm*	Phoxim	100 mg in 1 mL acetone, 4 µg/mL	Diet	L	Detoxification enzyme activity	Gastric juice spitting, body distortion, body shrinking, head and chest protrusion >activity of cytochrome P450 oxidase in the midgut and fat body <esterase activity in midgut, >in fat body >expression of cytochrome P450 oxidase genes, glutathione-S-transferase-related genes and esterase gene in midgut and fat body	Not evaluated	[304]
Lep	*Bm*	Phoxim	4 µg/mL	Diet	L	Larval midgut damage	Severe midgut damage and oxidative stress Beneficial effects of cerium Different gene expression regarding digestion and absorption Significant alterations of esterase, lysozyme, and amylase 48	Not evaluated	[331]
Lep	*Bm*	Phoxim	4.0 µg/mL phoxim solution	Diet	L	Adverse effects on the midgut	Head nystagmus, chaotic crawling, vomiting, cramps and paralysis Deaths of columnar cells and lysis of the goblet cells Abnormal cell structure, intestinal wall cracking and appearance of many sheet structures Changes in gene expression profile >activity of cytochrome P450s Apoptosis in the midgut Morphological changes in the nuclei and mitochondria Up-regulation of caspase-3 and CytC >protein levels of CytC in the total lysate and the cytosolic fraction <protein level of CytC in mitochondrial fraction Inhibition of the Toll signal pathway and the IMD signal pathway	Not evaluated	[318]
Lep	*Bm*	Phoxim Chlorpyrifos	Phoxim: 0.75–0.375 mg/L Chlorpyrifos: 1–0.5 mg/L	Diet	L	Expression analysis of peroxiredoxin family genes	>overall expression of peroxiredoxin family genes	Not evaluated	[319]
Lep	*Bm*	Phoxim	4 µg/mL phoxim solution	Diet	L	Symptoms Silk parameters Gene expression	Head nystagmus, chaotic climbing, vomiting, cramps, body shrinkage, paralysis <survival and cocooning Silk gland damage Oxidative stress Downregulation of the expression of silk-related genes Beneficial effect of TiO_2_ NPs	Not evaluated	[259]
Lep	*Bm*	Phoxim	4 µg/L	Diet	L	Symptoms Silk gland damage Gene expression Effects of CeCl_3_	Head nystagmus, chaotic climbing, vomiting, cramps, body shrinkage, paralysis <body weight, <survival <cocooning rate Damages to the silk gland, <protein content in the silk gland oxidative stress <antioxidant activities Differently expressed genes related mainly to silk protein synthesis, antioxidant capacity Beneficial effects of CeCl_3_	0.27 µg/g in silk gland 0.09 µg/g in silk gland with CeCl_3_	[245]
Lep	*Bm*	Phoxim	4 mg/mL	Diet	L	Nerve Toxicity and effects of TiO_2_ NPs	Gastric juice, spit, head nystagmus, body distortion, body shrinkage, paralysis and other symptoms <body weight and >mortality Histopathological changes in the brain Altered the release of neurotransmitters and the The activities of several important enzymes in the nerve conduction Oxidative stress Beneficial effects of TiO_2_ particles	Not evaluated	[290]
Lep	*Bm*	Chlorantraniliprole	2.5 ppm/L	Diet	L	Development Haemolymph	<body size, slow movement, body contraction, cessation of feeding, discolouration of body cuticle Loss of haemolymph <haemolymph volume with >age <protein level in fat body >urea levels >activity of aminotransferases	Not evaluated	[291]
Lep	*Bm*	Phoxim	4.0 μg/mL	Diet	L	Expression of genes in the fat body	774 differentially expressed genes Upregulation of expression of detoxification genes Downregulation of antimicrobial peptide genes Expression level of metamorphosis-related genes after exposure	Not evaluated	[320]
Lep	*Bm*	Phoxim	4 mg/L	Diet	L	Effects of TiO_2_ NPs + phoxim on transcription of antioxidant genes and enzyme activity in fat body	Differential expression of genes Upregulation of antioxidant genes transcriptional TiO_2_ NPs: >expression of the P450 family genes -->>fat body’s ability to metabolize phoxim and reduce phoxim-induced oxidative stress	Not evaluated	[321]
Lep	*Bm*	Phoxim	4 µg/mL	Diet	L	Expression profile analysis of P450 family genes	Head nystagmus, chaotic crawling, gastric juice spitting, cramps, and paralysis >mortality Changes in moulting hormone synthesis-related genes >expression of detoxification-related genes	Not evaluated	[260]
Lep	*Bm*	Phoxim	4.0 μg/mL	Diet	L	Body Weight, Survival, and Oxidative Stress Midgut damages	<body weight and survival rate >oxidative stress Midgut damages Differential expression of various genes	Not evaluated	[261]
Lep	*Bm*	Phoxim	1 µg/mL	Diet	L	Symptoms and mortality Oxidative stress parameters Effects on immune genes	Head nystagmus, chaotic crawling, gastric juice spitting, cramps and paralysis >mortality in the group treated with phoxim and nucleopolyhedrovirus >H_2_O_2_ and >levels of genes related to oxidative stress and immune response	Not evaluated	[262]
Lep	*Bm*	Phoxim	4 µg/mL	Diet	L	Nutrient metabolism and insulin signalling pathway in the midgut	Typical symptoms of poisoning <body weight >trypsin, lipase, and amylase activities Nutrition metabolism-related enzymes are dysregulated in the midgut <expression levels of insulin/insulin growth factor signalling (IIS) pathway genes	Not evaluated	[263]
Lep	*Bm*	Deltamethrin	0.025 µg/mL	Diet	L	Changes in gene expression	<expression of immune system functions genes at 30 °C Severe stress response <expression of peptidoglycan recognition protein genes, mucin, antimicrobial peptides genes <genes related to reactive oxygen species scavenging <mortality at >t°	Not evaluated	[308]
Lep	*Bm*	Imidacloprid Thiamethoxam Cypermethrin Deltamethrin Chlorpyrifos Acephate	5 mg/L	Diet	L	Mortality Toxicity Combined effects Toxicosis symptoms	>dose, <survival rate >exposure time, >toxicity Additive/antagonist effects based on a combination of compounds Stiffness of body, vomiting, <feed intake (with pyrethroid and organophosphates) Tremors, shortened body, vomiting, <feed intake (with neonicotinoids)	Not evaluated	[358]
Lep	*Bm*	Azadirachtin	1/2.5/5/7.5/10/15/20 mg/L	Diet	L	Rearing performances Effects on the prothoracic gland	>dose, >mortality >dose, no production of cocoon <size of cocoons Incomplete process of spinning cocoons and pupation <development of A, <cocooning rate Morphological changes and shrunken prothoracic glands Apoptosis in the prothoracic gland and DNA fragmentation <protein concentration in prothoracic glands <activities of Ca^2+^-Mg^2+^-ATPase >Ca^2+^ concentration	Not evaluated	[264]
Lep	*Bm*	Acetamiprid	0.01 mg/L	Diet	L	Symptoms and mortality Residue in haemolymph Gonad development Changes in the expression of 20E and JH-related genes	Mild poisoning symptoms <body and E weight Abnormalities in the ovaries and fallopian tubes <production of E Downregulation of ovarian development-related genes, ecdysone metabolism-related gene Upregulation of the expression of the juvenile hormone-related gene <ecdysone and >juvenile hormone in haemolymph	In haemolymph	[240]
Lep	*Bm*	Phoxim	5 µg	Injection	L	Silk gland damage Transcriptional response	Residue in the silk gland up to 24 h Upregulation of detoxifying enzymes-related genes	In silk glands up to 24 h	[239]
Lep	*Bm*	Imidaclorpid	0.6 mg/L	Diet	L	Intoxication and effects of N-acetyl-L-cysteine (NAC)	<growth, <survival rate, <food intake, <amount of digestion and ratio of digestion <cocoon mass, cocoon shell mass, ratio of cocoon shell >malondialdehyde >SOD enzyme activity <CAT activities <GSH-Px activities Beneficial effects of NAC	Not evaluated	[265]
Lep	*Bm*	Chlorantraniliprole Lambda-cyhalothrin Imidacloprid	0.1/1 mL/L	Diet	L	Pesticide toxicity	>mortality Different toxicity based on pesticides and time after exposure, different toxicity of a combination of compounds (additive effects)	Not evaluated	[266]
Lep	*Bm*	Azadirachtin	25 µg/pupa	Injection	P	Lipid transportation	Azadirachtin binds with LBD: interruption of protein–lipid interaction = suppression of lipid transportation to the ovary	Not evaluated	[353]
Lep	*Bm*	Chlorantraniliprole	0.01 mg/L	Diet	L	Growth and body mass Histochemical changes, Morphological structure Digestive enzyme activities Oxidative stress Differentially expressed genes (DEGs)	Head tremor, chaotic crawling, gastric juice spitting and cramps Stopped feeding, retarded movement, full-body contraction, yellowish epidermis <body mass Changes in morphology of midgut cells, <midgut cells, thinner stroma, disappearance of microvilli, karyopyknosis in the midgut Accumulation of ROS Swollen mitochondria, chromatin of the nucleus aggregated with a nonuniform distribution, and vacuoles in the cytoplasm Dysregulates the activities of digestive enzymes in the midgut <e expressions of oxidative phosphorylation pathway and antioxidant defence system related genes	Not evaluated	[267]
Lep	*Bm*	Lambda-cyhalothrin 2.5% (LAM) Chlorfenapyr 10% CLO Emamectin benzoate 5% EMA Profenofos 50% PRO Azadirachtin 0.03% AZA Fipronil 5% FIP Novaluron 10% NOV Dichlorvos DIC	LAM 1 mL/L CLO 1.5 mL/L EMA 0.4 gm/L PRO 1 mL/L AZA 2 mL/L FIP 0.75 mL/L NOV 0.5 mL/L DIC 2.63 mL/L	Diet	L	Toxicity Development Rearing performances	100% mortality with lambdacyhalothrin and emamectin benzoate Chlorofenapyr: less toxic Longer larval period <weights Delay in moulting <cocoon weight	Not evaluated	[268]
Lep	*Bm*	Chlorantraniliprole	0.01 mg/L	Diet	L	Detoxification enzyme activities Detoxification-related gene expression in the fat body	>activity of P450 and GST enzymes <CarE enzyme >expressions of the key genes in the PI3K/Akt/CncC signalling pathway and mRNA Inhibition of Akt at the protein level >detoxifying capability	Not evaluated	[322]
Lep	*Bm*	Novaluron	0.15 mL/L	Diet	L	Symptomatology Mortality Cell death analysis	Rupture in the integument, complete cessation of feeding, late development, incomplete ecdysis and production of defective cocoons >mortality (differences based on larval age) Cytotoxic effects Impaired development	Not evaluated	[269]
Lep	*Bm*	Acetamiprid	0.01 mg/L	Diet	L	Midgut damages	<size, <weight <cocoon mass, cocoon shell mass, ratio of cocoon-shell Histopathological and cellular microstructural changes in the midgut <activity of trypsin Differentially expressed genes involved in nutrient metabolism, stress responses, and inflammation pathways Oxidative stress	Not evaluated	[270]
Lep	*Bm*	Phoxim	1.0 μg/mL	Diet	L	Weight Gut microbial composition	<body and cocoon mass >bacterial community evenness Alteration of the structure of gut microbiota Differences in microbial communities <expressions of antimicrobial peptides >pathogenesis of *Enterobacter cloacae*	Not evaluated	[272]
Lep	*Bm*	Phoxim	4 μg/mL	Diet	L	Effects on the immune system	<body weight and survival rate Dysregulation of immune responses and expressions of immune-related genes in the midgut	Not evaluated	[271]
Lep	*Bm*	Acetamiprid	0.15 mg/L	Diet	L	Symptoms Silk gland damage	Yellowish colour <size Cocoon abnormalities <larval weight Residues in the posterior silk gland <cocoon weight, cocoon shell weight, ratio cocoon shell <silk glad size, morphological abnormalities Differently expressed genes <transcript levels of genes related to the synthesis of silk protein	Not evaluated	[241]
Lep	*Bm*	Chlorantraniliprole	0.01 mg/L	Diet	L	Effects on the silk gland Antioxidant and detoxification response	Slow movement, gastric juice spitting, the body was shrunk <body weight, thin-shelled cocoons, >mortality Damages of the posterior silk gland >activities and >expression of antioxidant and detoxification enzymes	Not evaluated	[273]
Lep	*Bm*	Pyriproxyfen	0.001/1/100 µg/L	Diet	L	Reproduction Gene expression	<number of E <hatch rate >dose, >damages <E size Affected absorption of nutrients, energy metabolism, ovary development and E formation	Not evaluated	[297]
Lep	*Bm*	Novaluron	0.15 mL/L	Diet	L	Silk gland Productive performances	Cytotoxic effects on epithelial cells of the silk gland Changes in the production of fibres and silk cocoons <weight of cocoon, defective cocoons	Not evaluated	[327]
Lep	*Bm*	Acetamiprid	0.15 mg/L	Diet	L	Detoxification enzymes in the midgut	>mortality + enlarged breast, head-shaking, gastric juice spitting, abdominal foot weakness, falling on mulberry leaves, body shrinking and softening, darker colour, anorexia, <body weight >CYP4M5, CYP6AB4, P450 enzyme activity <transcription levels of CarE, CarE-11 and the activity of CarE enzymes >transcription levels of GSTe3 and GSTd1, and GST enzyme activity >detoxification enzymes	In the midgut and haemolymph up to 96 h	[243]
Lep	*Bm*	Buprofezin Pymetrozine Flonicamid Dinotefuran Azadirachtin Dichlorovos	B: 1–2 mL/L P: 0.3–0.6 g/L F: 0.15–0.3 g/L DIN: 0.12–0.25 g/L A: 1–2 mL/L DIC: 1.32–2.63 mL/L	Diet	L	Reeling parameters	<length of filament Finer denier	Not evaluated	[328]
Lep	*Bm*	Pyriproxyfen	0.01 μg/L	Diet	L	Symptoms Body weight Silk parameters Gene expression	Gastric juice spitting, chaotic crawling and cramps <body weight <cocooning rate >rate of dead worm cocoons >cocoon weight <cocoon-shell ratio Histological changes in silk glands Downregulation of expression n levels of silk protein synthesis-related genes >expression levels of detoxification genes	Not evaluated	[275]
Lep	*Bm*	Pyriproxyfen	0.01 µg/mL	Diet	L	Immune signalling pathway and transcription of detoxification enzyme genes in the fat body	Cramps, chaotic crawling, and gastric juice spitting, <body weight, >cocoon mortality, <cocooning rate Histological changes in fat body: outflow of cellular content and appearance of some vacuoles in the cytoplasm Differential expressions of key genes in immune signalling pathways Upregulation of transcriptional levels of detoxification enzyme genes, and >activities of detoxification enzymes	Not evaluated	[274]
Lep	*Bm*	Phoxim	2.5 mg/L	Diet	L	Effects of Fe^2+^. Cu^2+^, Rb^2+^	Fe^2+^, Cu^2+^, Rb^2+^ alleviate the toxicity of phoxim about survival, ROS accumulation and oxidative stress	Not evaluated	[309]
Lep	*Bm*	Pyriproxyfen	0.001 mg/L	Diet	L	Midgut damages and related gene expression	Cellular damage to the midgut <expressions of digestive enzyme genes, oxidative phosphorylation genes, and antioxidant enzyme genes >activities of detoxification enzymes and the expressions of detoxification-related genes	Not evaluated	[323]
Lep	*Bm*	Guadipyr	5.25 and 10.50 mg/L	Diet	L	Microbiota Immune system	Alteration of midgut microbiota in terms of structure and richness Downregulation of antimicrobial peptide genes Immune disorders	Not evaluated	[332]
Lep	*Bm*	Acetamiprid	0.15 mg/L	Diet	L	Intestinal microbial homeostasis	ROS accumulation Dysregulation of intestinal immune signalling pathways Alteration of evenness and structure of the bacterial community Bacterial invasion in haemolymph <resistance to pathogens	Not evaluated	[310]
Lep	*Bm*	Novaluron	0.15 mL/L	Diet	L	Reproductive toxicity E production	Changes in organization, distribution, and Development of the cysts containing male germ cells Changes in morphological features of cell death <fertility of females, <of oviposition	Not evaluated	[298]
Lep	*Bm*	Chlorfenapyr	80/160/320/640/1280 mg/L	Diet	L	Transcriptional response of detoxifying enzyme genes	High number of differentially expressed genes, many related to detoxification pathways >mortality	Not evaluated	[276]
Lep	*Bm*	Fenvalerate	0.02 mg/L	Diet	L	Transcriptome Analysis in the Fat Body	Differentially expressed genes in the infected fat related to cellular components, molecular function and biological process	Not evaluated	[336]
Lep	*Bm*	Imidacloprid	20 mg/L	Diet	L	Mortality associated with lead contamination	Positive correlation with concentration and negative with the age of L of all 3-combination tested (imidacloprid formulation, imidacloprid technical grade and Pb)	Not evaluated	[292]
Lep	*Bm*	Glyphosate	4872/8663.78/15,406.62/27,397.27/48,719.10 mg/L	Diet	L	Growth Development Physiological functions	<growth <cocoon weight <feed digestibility <activities of alpha-amylase and trypsin Midgut damages Accumulation of peroxides in intestinal tissue >messenger RNA transcription of SOD and CAT	Not evaluated	[277]
Lep	*Bm*	Chlorantraniliprole	0.01 mg/L	Diet	L P	Impact on epidermis during PP-P transition	Disruption of energy homeostasis, oxidative stress, autophagy and apoptosis in the epidermis >trehalose <trehalose metabolism genes <chitin content	Not evaluated	[287]
Lep	*Bm*	Chlorantraniliprole	0.001 mg/L 2.783/7.746/21.558/60/166.989/464.758 × 10^−3^ mg/L	Diet	L	Mechanism of autophagy	<Ca^2+^-ATPase and CA^2+^-Mg^2+^-ATPase activities Activation of AMPK-related genes AMPK-a and AMPK-b Upregulation of autophagy-related genes	Not evaluated	[338]
Lep	*Bm*	Dimethoate	25, 50, and 100 ppm	Diet	L	Oxidative stress DNA damage Histological alterations	Weight loss Damage at the histological level to the mid-gut, silk gland, and fat body <level of antioxidants like CAT, SOD, GPx, GSH, GS Lipid peroxidation in the silk gland, gut, and fat body	Not evaluated	[278]
Lep	*Bm*	Dinotefuran	0.4 mg/L (LCG) 4 mg/L (HCG)	Diet	L	Biochemical toxicity Transcriptome aberration	>dose, >mortality; <larval weights Intoxication (antifeeding, vomit, softening of the body) (HCG) >activity of GST and UGT enzymes in fat body (LCG) >activity of GST, P450, CarE and UGT enzymes in fat body (HCG) >activity of GST enzyme in midgut (HCG) Upregulation of detoxifying enzymes genes >energy metabolism, oxidative stress, detoxification <carbon metabolism, fatty acids biosynthesis, pyruvate metabolism, citrate cycle Activation of MAPK/CREB, CncC/Keap1, PI3K(Akt and Toll/IMD pathways	Not evaluated	[279]
Lep	*Bm*	Chlorantraniliprole	0.01 mg/L	Diet	L	Autophagy and apoptosis	Release of intracellular Ca^2+^ in BmN cells Induction of Ca^2+^-dependent genes in midgut Damaged mitochondria, autophagosomes, nuclear membrane rupture and condensed chromatin Downregulation of genes in the oxidative phosphorylation pathway Upregulation of autophagy-related genes and apoptosis genes >protein levels of LC3-II and ATG7, gene caspase 3	Not evaluated	[333]
Lep	*Bm*	Fenvalerate DDVP	Fenvalerate 0.05 µg/L DDVP8 µg/L	Diet	L	Expression levels of seven reference genes (RGs)	Different levels of stability of stress-responsive genes based on the evaluated tissue and type of pesticide	Not evaluated	[337]
Lep	*Bm*	Dimethoate	200 mg/L	Diet	L P A	Oviposition, fertility, reproduction	<moths >number of E inside female genitalia, <E laid, >production of unhealthy E Morphological changes in cocoons and P Slower development Differentially expressed genes related to promoting trehalase transporter genes, stress response genes, zinc fingers protein genes, epidermal protein genes and 5-HT pathway related genes	In E	[242]
Lep	*Bm*	Imidacloprid Thiamethoxam	0.125/0.25/0.50/1/2 mg/L	Diet Spraying	L	Development Body Weight Economic Characteristics	Different LD50 based on method of application >developmental time wth >dose <sputum production <weight with >dose (spraying) >CarE and GST activities >expression of CarE-11, GSTe3, GSTz2 genes DNA damage	Not evaluated	[280]
Lep	*Bm*	Chlorantraniliprole	0.01 mg/L	Diet	L	Apoptosis in the silk gland	Abnormal silk gland development release of intracellular Ca^2+^, triggering of Ca^2+^-dependent gene transcription <oxidative phosphorylation and antioxidant enzyme-related genes in silk gland (peroxide accumulation) >autophagy-related genes and protein levels of LC3-I and LC3-II	Not evaluated	[339]
Lep	*Bm*	Dinotefuran Imidaclorpid Methoprene Fenoxycarb	Dinotefuran: 4 mg/L Imidaclorpid: 0.8 mg/L Methoprene: 1 mg/mL Fenoxycarb: 3 ng/L	Diet (dinotefuran, imidaclorpid, fenoxycarb) Topic application (methoprene)	L	Expression of the MyD88 gene	>expression of MyD88 gene in fat body (dinotefuran, methoprene, fenoxycarb) and in fat body and midgut (imidacloprid)	Not evaluated	[324]
Lep	*Bm*	Pyriproxyfen	1 × 10^−4^ mg/L	Diet	L	Autophagy and apoptosis in silk gland (PSG)	Shrinkage, vacuolization, and fragmentation in the posterior silk gland Induction and apoptosis in the PSG gland by autophagy-related genes Activation of juvenile hormone signalling pathway genes Inhibition of 20-hydroxyecdysone (20E) signalling pathway genes Upregulation of autophagy and apoptosis-related genes	Not evaluated	[330]
Lep	*Bm*	Indoxacarb enantiomers (enantiopure R-indoxacarb, enantiopure S-indoxacarb and enriched S-indoxacarb)	enantiopure R-indoxacarb 2.164 µg/mL enantiopure S-indoxacarb 0.0392 µg/mL enriched S-indoxacarb 0.1378 µg/mL	Diet	L	Toxicity, bioaccumulation and biotransformation	No chiral transition from S-indoxacarb to R-indoxacarb Indoxacarb bioaccumulated in L >bioconversion in metabolite DCJW by S-indoxacarb S-indoxacarb >toxic than R-indoxacarb S-indoxacarb: <weight gain <Food intake	Not evaluated	[281]
Lep	*Bm*	Dinotefuran	0.4–4 mg/L	Diet	L	Autophagy and apoptosis	>reactive oxygen species (ROS) and malondialdehyde (MDA) in midgut and fat body (oxidative stress) Alteration of autophagy and apoptosis genes >expression levels of autophagy/apoptosis-related proteins	Not evaluated	[340]
Lep	*Bm*	λ-cyhalothrin	0.13, 0.21, 0.27, 0.45 mg/L	Diet	L	Sublethal toxicity and transcriptome-wide biological changes	Food refusal, sluggish movement, head tilted back, and vomiting of intestinal fluid <body size Slow recovery of size after stopping exposure <body weight P malformation Differential gene expression <oxidative phosphorylation pathway	Not evaluated	[282]
Lep	*Bm*	Spinosad	0.1, 0.05, and 0.025 ppm	Diet	L	Bioassay and expression alterations of acetyl cholinesterase enzyme gene	0.1 ppm: >mortality Morphological symptoms (vomit, lying on the side, body shrinking >expression levels of Ace gene (>expression with >dose)	Not evaluated	[306]
Lep	*Bm*	Nitenpyram	4/6/8/10 mg/L 0.125/0.2/0.5/1/2/4 mg/L 5/10/15/20/25/30 mg/L Based on the formulation of reagents	Diet	L	Toxicity	Vomiting, anti-feeding, head shaking, body shrinking and softening and dysplasia Soft and black body Oxidative stress DNA damage Changes in gene patterns	Not evaluated	[307]
Lep	*Bm*	Fenpropathrin (FPP)	0.5, 1, 2, 4, 8 μg/mL 0.03, 0.06, 0.125, 0.25, 0.5 μg/mL	Diet	L	Metabolites	>dose, >mortality (100% with 8 µg/mL) 26 of 27 metabolites showed significant differences	Not evaluated	[284]
Lep	*Bm*	Tolfenpyrad	2.5 mg/L	Diet	L	Development and response mechanism	Mild symptoms of intoxication <body weight, <feeding, slow crawling, >development duration <cocooning rate, <eclosion rate, <pupation rate Differential gene expression related to xenobiotics degradation and metabolism, the immune system and the digestive system >abundance of Enterobacter and Staphylococcus <abundance of Tyzzerella and Methylobacterium-Methylorubrum	Not evaluated	[285]
Lep	*Bm*	λ-cyhalothrin	0.21 mg/L	Diet	L	JH degradation pathway	Longer developmental time >JH III titer in haemolymph Upregulation of JH interacting genes Downregulation of 13 JH degradation genes in the midgut Inhibitory effects on JH esterase, JH epoxide hydrolase, and JH diol kinase	Not evaluated	[334]
Lep	*Bm*	Broflanilide	0.011 mg/L	Diet	E L P A O	Development and detoxification mechanism	Gastric juice spitting, anal prolapse, and convulsions Deformities in P and A <larval and pupal weight <cocooning rate, good cocoon rate, eclosion rate <fecundity, number of E, hatching rate Worse silk parameters >ROS and oxidative stress in the midgut >antioxidant enzymes activity Activation of innate immune signalling pathways and AMP genes <survival rate of O at all stages	Not evaluated	[283]
Lep	*Bm*	Fenpropathrin (FPP)	2.5 µg/mL	Diet	L	Toxicity of pesticides in nano/microplastics (NMPs)	<survival and growth, delayed development, <cocoon production (FPP + NMPs) Physical and oxidative damage to the midgut Altered gene expression related to juvenile hormone (JH) and silk protein synthesis Changes and <of beneficial bacteria in gut microbiome	Not evaluated	[286]
Lep	*Bm*	Novaluron	0.001 mg/L	Diet	L	Oviposition Expression of Ovarian Development Related Genes	<number of E <hatch rate <fertilization rate Slower development of germ cells <number of oocytes and oogonia <expression of Vg, Ovo gene, Otu gene, Sxl-L gene, EcR gene and JHBP2 gene	Not evaluated	[299]
Lep	*Bm*	Phoxim	0.013/0.032/0.079/0.316 µg/ml	Diet	L	Silk production Endocrine system	Silk gland damage >malondialdehyde, >SOD and POD activities <juvenile hormone <expression of fibroin synthesis gene	Not evaluated	[329]

Dip: Diptera; Col: Coleoptera; Ort: Orthoptera; Lep: Lepidoptera; *Hi*: *Hermetia illucens*; *Md*: *Musca domestica*; *Adi*: *Alphitobius diaperinus*; *Tm*: *Tenebrio molitor*; *Lm*: *Locusta migratoria*; *Ado*: *Acheta domesticus*; *Ga*: *Grillus assimilis*; *Bm*: *Bombyx mori*; E: eggs; L: larvae; N: nymphs; P: pupae; A: adults; O: offspring.

## Data Availability

The data represent the original findings of this study and are available within the article.
